# Ribosomal expansion segment contributes to translation fidelity via N-terminal processing of ribosomal proteins

**DOI:** 10.1093/nar/gkaf448

**Published:** 2025-05-28

**Authors:** Riku Nagai, Olivia L Milam, Tatsuya Niwa, William J Howell, Jacob A Best, Hideji Yoshida, Carver D Freeburg, John M Koomen, Kotaro Fujii

**Affiliations:** Center for NeuroGenetics, University of Florida, Gainesville, FL 32610, United States; Department of Molecular Genetics and Microbiology, University of Florida, Gainesville, FL 32610, United States; Center for NeuroGenetics, University of Florida, Gainesville, FL 32610, United States; Department of Molecular Genetics and Microbiology, University of Florida, Gainesville, FL 32610, United States; Cell Biology Center, Institute of Integrated Research, Institute of Science Tokyo, Yokohama, Kanagawa 226-8503, Japan; Center for NeuroGenetics, University of Florida, Gainesville, FL 32610, United States; Department of Molecular Genetics and Microbiology, University of Florida, Gainesville, FL 32610, United States; Center for NeuroGenetics, University of Florida, Gainesville, FL 32610, United States; Department of Molecular Genetics and Microbiology, University of Florida, Gainesville, FL 32610, United States; Department of Physics, Osaka Medical and Pharmaceutical University, Takatsuki, Osaka 569-8686, Japan; Center for NeuroGenetics, University of Florida, Gainesville, FL 32610, United States; Department of Molecular Genetics and Microbiology, University of Florida, Gainesville, FL 32610, United States; Department of Molecular Oncology, Moffitt Cancer Center, Tampa, FL 33612, United States; Center for NeuroGenetics, University of Florida, Gainesville, FL 32610, United States; Department of Molecular Genetics and Microbiology, University of Florida, Gainesville, FL 32610, United States

## Abstract

Eukaryotic ribosomes exhibit higher mRNA translation fidelity than prokaryotic ribosomes, partly due to eukaryote-specific ribosomal RNA (rRNA) insertions. Among these, expansion segment 27L (ES27L) on the 60S subunit enhances fidelity by anchoring methionine aminopeptidase (MetAP) at the nascent protein exit tunnel, accelerating co-translational N-terminal initiator methionine (iMet) processing. However, the mechanisms by which iMet processing influences translation fidelity remain unknown. Using yeast *in vitro* translation (IVT) systems, we found that inhibiting co-translational iMet processing does not impact ribosome decoding of ongoing peptide synthesis. Instead, our novel method to monitor iMet processing *in vivo* revealed that ribosomes purified from strains lacking MetAP ribosomal association (ES27L *Δb1-4*) or major yeast MetAP *(Δmap1*) increase iMet retention on ribosomal proteins (RPs). Given the densely packed structure of ribosomes, iMet retention on RPs may distort ribosomal structure and impair its function. Indeed, reconstituted IVT systems containing iMet-retaining ribosome subunits from ES27L *Δb1-4* strain, combined with translation factors from wild-type strains, elucidated that iMet retention on the 40S ribosomal subunit causes translation errors. Our study demonstrated the critical role of ES27L in adjusting ribosome association of universally conserved MetAP enzyme to fine-tune iMet processing of key RPs, thereby ensuring the structural integrity and functional accuracy of eukaryotic ribosomes.

## Graphical Abstract

**Graphical Abstract:**
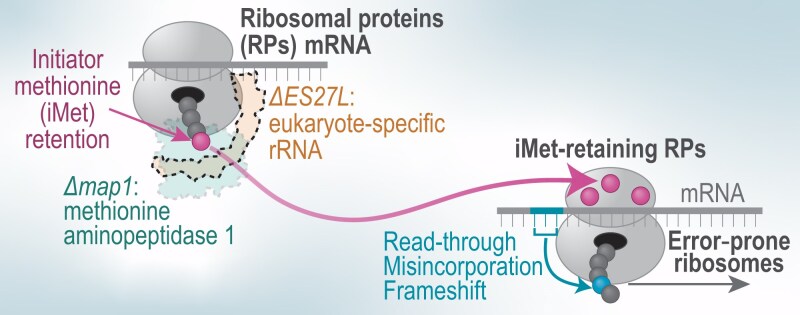

## Introduction

Messenger RNA (mRNA) translation is a highly complex process, centered on the ribosome, which is composed of 80 ribosomal proteins (RPs) and 4 ribosomal RNAs (rRNAs) in Eukaryotes. The ribosome requires coordinated action with various translation factors (TFs) and ribosome-associated proteins (RAPs) to orchestrate the multistep process of mRNA translation, ensuring the accurate synthesis of proteins [1–3]. Longer proteins require more robust proofreading mechanisms, and indeed, there is a correlation between mean protein length and translation fidelity in *Escherichia coli*, *Saccharomyces cerevisiae*, and *Homo sapiens* [4–6]. Interestingly, the size and complexity of the ribosome also increase from *E. coli*, *S. cerevisiae*, to *H. sapiens*, even though the core function of the ribosome, particularly the translation elongation step, is highly conserved across species [7–9]. In *E. coli*, the ribosome has a mass of ∼2.3 MDa, compared to 3.3 MDa in *S. cerevisiae* and 4.3 MDa in *H. sapiens*. This increase in size is largely attributed to the insertion and expansion of eukaryote-specific rRNA domains, known as expansion segments (ESs) [10]. These ESs surround the conserved core structure of the ribosome. Recent studies have shown that these domains are not merely mutations at nonessential regions of rRNA but the result of selection due to their functions, such as transcript specific translation, increasing fidelity of mRNA translation, and recruitment of RAPs on the ribosomes [11–15].

Previous research, including our own, demonstrated the function of the longest ES, ES27L in 25S rRNA on the 60S ribosomal subunit, in the fidelity of mRNA translation and maintenance of protein homeostasis in *S. cerevisiae* [11, 12]. ES27L is a flexible domain within the yeast ribosome [16], and its length and sequence vary between species. While the ancestral structure of ES27L in *E. coli* is a 33 nt short stem-loop, the domain has expanded to 159 nt in *S. cerevisiae* and over 700 nt in *H. sapiens*. As a result, mammalian ES27L forms a tentacle-like structure with variable sequences between organisms [9, 17, 18]. The deletion of the distal flexible portion of ES27L (ES27L *Δb1-4*) (Supplementary Fig. S1A) in yeast reduced translation fidelity and increased stop codon readthrough, amino acid (AA) misincorporation, and ribosomal frameshifting [11]. Such deletion of ES27L also affects protein homeostasis and increases cellular protein aggregation [12]. Furthermore, multicellular organisms are remarkably sensitive to translation errors [19, 20]. Increasing AA misincorporation in mice increases unfolded proteins, which induces protein aggregation and neurodegeneration [21]. Constant production of error-prone ribosomes significantly impacts protein homeostasis over time, thereby accelerating aging and also fostering neurodegeneration in mice [22, 23]. Therefore, maintaining accurate ribosomal decoding is critical for protein homeostasis and lifespan [24–26].

**Figure 1.**
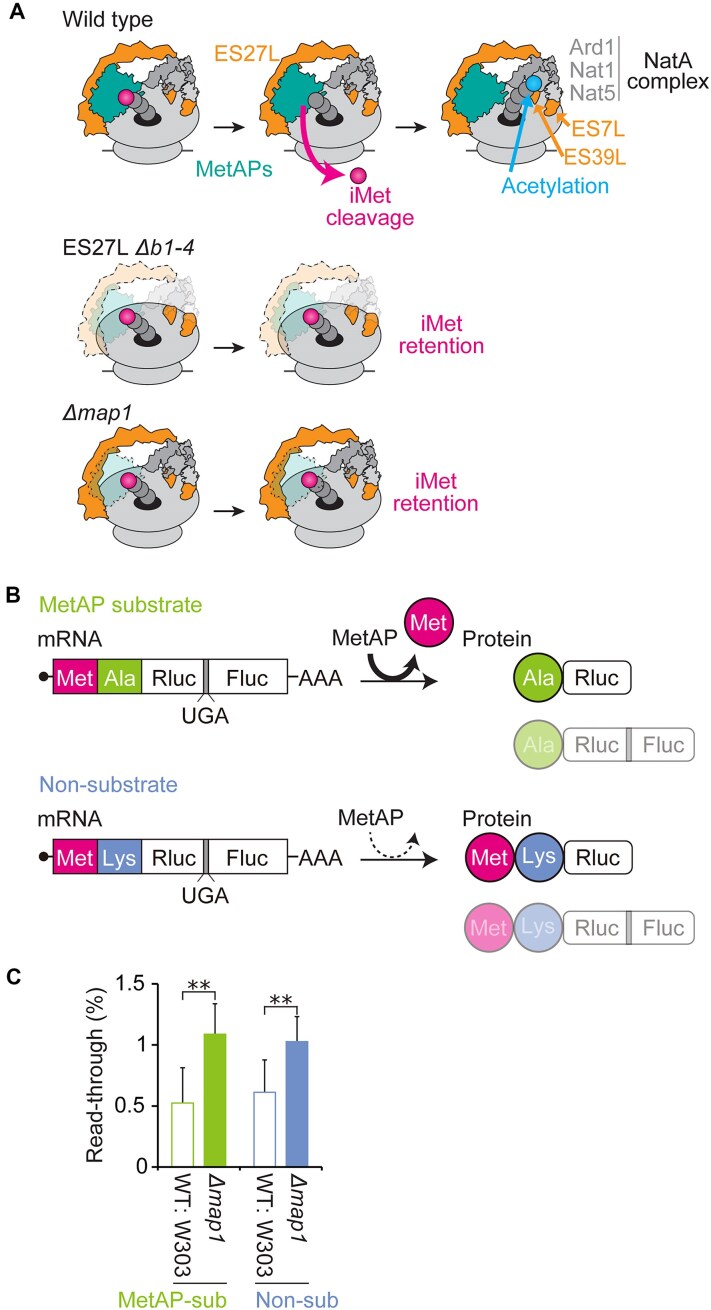
Initiator methionine (iMet) processing by MetAPs regulates mRNA translation fidelity even for the MetAP nonsubstrate protein synthesis. (**A**) Schematic of co-translational N-terminal processing of wild-type (top), ES27L *Δb1-4* (middle), and *Δmap1* (bottom) strains. ES27L allows MetAP (Map1 in yeast) to associate with the ribosome. After Map1 cleaves initiator methionine (iMet) from the nascent polypeptide, the NatA acetylates the second AA. NatA is composed of the catalytic subunit Ard1, the scaffold Nat1, and the auxiliary subunit Nat5. (**B**) Schematic of reporter constructs: The MetAP substrate reporter has alanine (Ala) as the second AA. The iMet in the substrate construct will be co-translationally processed by MetAP. The MetAP nonsubstrate reporter has lysine (Lys) as the second AA, which will retain iMet on protein products. Both constructs contain a UGA stop codon between Renilla (Rluc) and Firefly (Fluc) luciferase. Accurate mRNA translation produces only Rluc. Stop codon readthrough produces Rluc-Fluc. (**C**) The percentage of stop codon readthrough rates of MetAP substrate and nonsubstrate constructs was measured in the WT: W303 and *Δmap1* strains. To measure mistranslation efficiency, normalized Fluc activities were further normalized to the ‘‘wild-type’’ construct, which does not have an insertion of the stop codon between Rluc and Fluc for each MetAP substrate and nonsubstrate construct. Data are presented as mean + SD (*t*-test, **P < 0.01; *n* > 3).

To understand the molecular mechanism of fidelity control by ES27L, we previously showed that ES27L anchors methionine aminopeptidases (MetAPs) directly on the ribosome [11]. Ribosome structure studies further revealed that ES27L holds MetAPs and its homologs close to the nascent peptide exit tunnel [27–30]. MetAP cleaves off the initiator methionine (iMet) from ∼50% to 70% of all nascent proteins as a part of N-terminal processing for newly synthesized proteins [31]. MetAP selects substrates with small and uncharged second AA [31]. Eukaryotes have two MetAP genes (*MAP1* and *MAP2* in yeast), and the double knockout of these genes is lethal in yeast [32]. Ribosomes in ES27L *Δb1-4* yeast strain (Supplementary Fig. S1A) cannot efficiently recruit either Map1 or Map2 proteins, which leads to decreased iMet processing efficiency and reduced mRNA translation fidelity [11]. Similarly, the deletion strain of the major yeast MetAP (Δ*map1*) also exhibits reduced mRNA translation fidelity. Given that the catalytically dead Map1 protein could not rescue the reduced fidelity, MetAP activity and iMet processing have a crucial role in mRNA translation fidelity [11]. In mammalian cells, treatment with the MetAP inhibitor, bengamide B, increases iMet retention and translation errors, supporting the role of MetAP activity for translation fidelity and exemplifying the conserved function of MetAP for translation fidelity in mammals. Thus, the crucial role of ES27L in translation fidelity appears to lie in its ability to hold MetAPs close to their substrate, iMet, at the exit tunnel, thereby enhancing co-translational iMet processing (Fig. 1A) [11]. However, underlying molecular mechanisms of how iMet processing contributes to translation fidelity remain unknown. Understanding these mechanisms will provide novel insight into outstanding questions of how species-specific translation fidelity programs are established across evolution.

MetAP is one of the RAPs that associate with the most flexible domain of the ribosome, ES27L. Many more RAPs, also involved in co-translational processing, interact with ES27L, making it a central hub for co-translational processing. As the nascent peptide emerges from the exit tunnel, it is recognized by the nascent polypeptide-associated complex (NAC) and undergoes iMet cleavage followed by the acetylation of the N-terminal AA [33, 34]. N-terminal AAs are acetylated by an N-terminal acetyltransferase complex (Nat A or B), which is also associated with ES27L, as well as with ES7L and ES39L (Fig. 1A) [30, 35]. While iMet processing is a conserved and essential process across all domains of life, even seen in mitochondria and chloroplasts [36, 37], N-terminal acetylation is more abundant in eukaryotes, and Nat complexes do not exist in bacteria. In yeast and human cultured cells, N-terminal acetylation is linked to protein homeostasis [38, 39], which might be contributing to the reduced protein homeostasis observed in the ES27L mutant strain [12]. Beyond N-terminal processing, ES27L also interacts with chaperones involved in co-translational protein folding, such as the ribosome-associated complex (RAC) [40], which is known to play a role in translation fidelity [41]. Interestingly, a structural study demonstrated that RAC bridges nascent proteins exiting at the 60S with ES12S on the 40S small ribosomal subunit [42]. Thus, RAC can regulate translation fidelity by promoting feedback between nascent proteins and the decoding center [43].

Therefore, as a molecular basis for ES27L in fidelity regulation, we initially hypothesized that co-translational iMet processing might influence ribosomal decoding of “ongoing” peptide synthesis. However, our research, leveraging genetic modification of reporter constructs and *in vitro* translation (IVT) systems from yeast extract, denied this hypothesis and elucidated a novel molecular mechanism of fidelity regulation through iMet processing. To our surprise, we demonstrated that co-translational iMet processing is not the mechanism of fidelity control. Instead, iMet retention on cytoplasmic proteins affects translation fidelity. To further understand the molecular mechanisms behind this phenomenon, we developed a novel technique to identify iMet-retaining proteins *in vivo* and revealed that ribosomes with reduced iMet processing in mutant yeast strains exhibited selective iMet retention on RPs. Leveraging reconstitution of the IVT system, we demonstrated that those iMet-retaining ribosomes were more prone to translation errors. Gene ontology (GO)-term analysis further indicated that RPs are conserved substrates of MetAPs, with >70% of eukaryotic large subunit RPs being MetAP substrates, compared to only 40% of large subunit RPs in bacteria. These findings emphasize the important role of eukaryote-specific ES27L in recruiting MetAP to the ribosome to enhance iMet processing, highlighting an evolutionary strategy to produce tightly packed, highly accurate ribosomes in eukaryotes.

## Materials and methods

### Yeast strains and culture

Yeast strains, *Saccharomyces cerevisiae*, used in this paper are listed in Supplementary Table S1. Since the genetic backgrounds of ES27L *Δb1-4* and *Δmap1* strains are different, we used WT_rRNA as the counterpart wild-type strain for ES27L *Δb1-4* and WT: W303 as the counterpart wild-type strain for the *Δmap1* strain. For yeast growth, YPAD medium (10 g/l yeast extract, 20 g/l peptone, 40 mg/l adenine sulfate, and 20 g/l glucose) or synthetic dextrose (SD) medium (6.7 g/l yeast nitrogen base and 20 g/l glucose plus appropriate AAs drop out mix) was used.

### Plasmid construction

Plasmids used in this paper are listed in Supplementary Table S2. Stop codon readthrough reporters for MetAP substrate and nonsubstrate were cloned by site-directed mutagenesis for the second AA to Ala (GCT) or Lys (AAA), respectively, of Rluc in pJD375 and pJD433 [44]. IVT reporters encode NanoLuc luciferase (NLuc) with a FLAG tag at the N-terminus and an HA tag at the C-terminus that was inserted into the pRS316 plasmid [45] using SpeI and XhoI restriction sites. The UGA stop codon was substituted with the 88th histidine of NLuc as a stop codon readthrough construct. To monitor iMet retention *in vivo*, Nluc with a FLAG tag at the C-terminus was used, where all Met codons within the ORF, except for iMet, were replaced with Thr (ACG), and the second AA in the ORF was substituted with either a MetAP substrate [Ala (GCT), Ser (TCT), or Gly (GGT)] or a nonsubstrate (Lys (AAA)).

### *In vivo* translation fidelity reporter assay

Stationary yeast cultures transformed with translation fidelity constructs were diluted to OD_600_ = 0.08 and grown to the log phase (OD_600_ = 0.8). For bengamide B (Santa Cruz, sc-397521A) treatment, the cell culture at OD_600_ = 0.8 was further diluted to OD_600_ = 0.2, and the bengamide B was added into the medium. The culture was then incubated until reaching an OD _600_ = 1.2 to harvest. Collected yeast pellets were lysed by passive lysis buffer and assayed using the Dual-Luciferase Reporter Assay System (Promega). To assess mistranslation efficiency *in vivo*, Rluc and Fluc tandem reporters were used and Fluc activity was normalized to Rluc (Fluc/Rluc) to account for input variability. The error rate, expressed as a percentage, was calculated by dividing the Fluc/Rluc ratio from the stop codon construct by the ratio from the wild-type construct without a stop codon.

### Translation fidelity assay in mouse embryonic stem cells

Stop codon readthrough constructs were transfected by Lipofectamine 2000 (Invitrogen) in 24-well plates. For bengamide B (Santa Cruz) treatment, the drug (0.1 μM) was added into the medium 2 h after transfection. Cells were then incubated for 3, 12, or 24 h. Then cells were harvested by trypsin and assayed using the Dual-Luciferase Reporter Assay System (Promega).

### *In vitro* translation fidelity assay

Yeast cell lysate was prepared as described [46], with minor modifications. Specifically, the lysate was prepared from 6 L of yeast culture at an OD_600_ of 1.0. Cells were resuspended in the designated buffer at a ratio of 0.3 ml per gram of cells. All buffers contained complete Ethylenediaminetetraacetic acid (EDTA) free protease inhibitor cocktail tablets (Roche). The template for *in vitro* transcription was prepared by PCR using IVT reporter plasmids as a template with specific primer sets (see Supplementary Tables S3 and S4 for the sequences of the primers and templates, respectively). The m7G Cap was added during *in vitro* transcription using HiScribe T7 ARCA mRNA Kit (NEB) followed by poly(A)-tailing and purification using the RNA Clean & Concentrator (Zymo Research).

The standard translation reaction mixtures (10 μl) contained 30 mM Hepes-KOH (pH 7.4), 100 mM KOAc, 6 mM Mg(OAc)_2_, 2 mM Dithiothreitol (DTT), 0.01 mM of each AA, 1 mM ATP, 0.1 mM GTP, 20 mM creatine phosphate, 0.06 units/ml creatine kinase, 0.4 units/ml RNaseOUT Recombinant Ribonuclease Inhibitor (Invitrogen), 0.1 mM mRNA, and yeast cell lysate at a final protein concentration of 10 mg/ml. In the ribosome-switching IVT system, 0.2–0.75 μM ribosomal fraction and TFs at a final protein concentration of 4 mg/ml replaced the lysate. For the Δ*map1* strain, Mg(OAc)_2_ was adjusted to 1 mM to enhance ribosome activity, with the control WT: W303 ribosomes tested similarly. Ribosome concentrations were adjusted to ensure comparable activity between strains (WT: W303 as 0.2 μM rRNA and *Δmap1* as 0.5 μM). In the 40S and 60S IVT system, the 40S and 60S subunits were each added at a final concentration of 1.1 μM, along with the top fraction from the sucrose density gradient (2.6 mg/ml final protein concentration) and TFs (4 mg/ml final protein concentration). Drugs like paromomycin or bengamide B were added to the WT: W303 IVT lysate as needed. Translation reactions were incubated for 2 h at 21°C, quenched, diluted 10-fold with passive lysis buffer, and analyzed using the Nano-Glo Dual-Luciferase Reporter Assay System (Promega, N1610). The wild-type Nluc mRNA served as a control to assess baseline translation activity across IVT batches. The error rate was calculated by the Nluc activity of error-detecting mRNAs (e.g. stop codon or frameshift) normalized to the wild-type mRNA.

### Separation of ribosome and translation factors from yeast cell lysate for IVT

The yeast cell lysate was centrifuged to separate ribosomes and TFs, as reported [47]. Ribosome concentrations were calculated assuming that 1 *A*_260_ unit (40 ng/μl RNA) corresponds to 20 nM for 80S ribosomes.

### Ribosome subunit separation for IVT

The purified, tightly coupled 80S ribosomes were dissociated into subunits by incubation at 37°C for 10 min in a buffer containing 50 mM HEPES-KOH (pH 7.4), 500 mM KCl, 2 mM MgCl_2_, and 2 mM DTT, supplemented with 1 mM puromycin [48]. An amount of the puromycin treated ribosomes equivalent to 1.5 mg of RNA was then loaded onto 5%–20% sucrose gradients [50 mM Hepes-KOH (pH 7.4), 500 mM KCl, 5 mM MgCl_2_, 0.1 mM EDTA, and 2 mM DTT] and centrifuged at 47 000 *g* (Beckman SW28Ti 19, 000 rpm) for 16 h at 4°C. The top fraction containing RAPs, along with the 40S and 60S fractions, was collected separately. Each fraction was concentrated and simultaneously buffer-exchanged into 20 mM HEPES-KOH (pH 7.4), 100 mM KOAc, 2 mM Mg (OAc)_2_, and 2 mM DTT using Amicon Ultra centrifugal filters (MWCO 3 kDa for the top fraction; 100 kDa for the 40S and 60S fractions). Ribosome concentrations were calculated assuming that 1 *A*_260_ unit (40 ng/μl RNA) corresponds to 60 nM for 40S and 30 nM for 60S ribosomes.

### Gene ontology term analysis

GO term enrichment analysis was performed on putative MetAP substrates in *E. coli*, *S. cerevisiae*, and *Mus musculus*. Putative MetAP substrates were identified by parsing GFF3-annotated FASTA files for protein-coding genes with methionine as the first AA and one of the following small or uncharged AAs as the second: alanine (Ala), cysteine (Cys), glycine (Gly), serine (Ser), threonine (Thr), and valine (Val) [31]. GFF3 and FASTA files from Ensembl were used for *E. coli* MG1655 and *S. cerevisiae* S288c as representative of the *E. coli* and *S. cerevisiae* genomes, respectively. The basic gene annotation (CHR) GFF3 and protein-coding transcript sequences (CHR) FASTA files for M36 (GRCm39) from Gencode were used as representative of the *M. musculus* genome. GO term enrichment analysis was performed on the identified MetAP substrates using the PANTHER 19.0 Overrepresentation Test [49, 50] with the default gene reference list for each species. GO terms with a fold enrichment and a false discovery rate (FDR) *P*-value of < 0.05 were considered significant.

### Monitoring iMet retention using reporter

Stationary yeast cultures were diluted to OD_600_ = 0.08 and grown to the log phase (OD_600_ = 0.8) in SD media without methionine, with 50 μM L-Homopropargylglycine (HPG) (Vector Laboratories) added instead. Harvested cells were lysed in liquid nitrogen using a mortar and pestle. The resulting cell powder was resuspended in lysis buffer [20 mM Hepes-KOH (pH 7.4), 100 mM KOAc, 2.5 mM Mg (OAc)_2_, 0.5 mg/ml heparin, 2 mM DTT, and complete EDTA-free protease inhibitor cocktail tablets (Roche)] at a ratio of 0.3 ml per gram of cells. The lysate was centrifuged at 1300 *g* for 10 min at 4°C. The resulting supernatant was further centrifuged at 12 000 *g* for 10 min at 4°C and the supernatant was collected. Cell lysate obtained from 250 ml of HPG-incorporated yeast culture was incubated with 50 μl of ANTI-FLAG M2 affinity Gel (Millipore) rotating for 1 h at 4°C. Beads were washed with 100 column volumes of IP (immunoprecipitation) buffer [50 mM Tris–HCl (pH 7.4), 150 mM NaCl, 2.5 mM MgCl_2_, and 1% (v/v) Triton X-100] and eluted with IP buffer containing 100 μg/ml FLAG peptide.

### Ribosome purification and subunit separation for click reaction

Cell lysate obtained from 500 ml of HPG-incorporated yeast culture was layered onto an equal volume of sucrose cushion [20 mM Hepes-KOH (pH 7.4), 100 mM KCl, 20 mM MgCl_2_, 1 M sucrose, 0.5 mg/ml heparin, 2 mM DTT, and 0.1 mM Phenylmethylsulfonyl fluoride (PMSF)) and centrifuged at 287 000 *g* (Beckman TLA110 84 000 rpm) for 1 h at 4°C [51]. To dissociate individual subunits, the resulting ribosomal pellet was resuspended in subunit separation buffer [50 mM Hepes-KOH (pH 7.4), 500 mM KCl, 2 mM MgCl_2_, and 2 mM DTT] with 1 mM puromycin, and incubated for 10 min at 37°C [48]. An amount of the puromycin treated ribosomes equivalent to 500 μg of RNA was then loaded onto 5%–20% sucrose gradients [50 mM Hepes-KOH (pH 7.4), 500 mM KCl, 5 mM MgCl_2_, 0.1mM EDTA, and 2 mM DTT] and centrifuged at 47 000 *g* (Beckman SW41Ti 19 600 rpm) for 16 h at 4°C [48]. Separated subunits were obtained using a fractionator with continuous *A*_260_ measurement, and the buffer was exchanged to click compatible polysome dissolving buffer [30 mM Hepes-KOH (pH 7.4), 100 mM NaCl, and 5 mM MgCl_2_] during concentration with Amicon concentrators (100 kDa MWCO).

### Click reaction and detection of HPG-labeled protein by SDS–PAGE

The FLAG-IP eluate from 250 ml of yeast culture or an amount of ribosomes equivalent to 1 μg of RNA was mixed with a reagent mix (5.5 mM sodium ascorbate, 1% SDS, 1.5 mM CuSO_4_, 2.5 mM tris-hydroxypropyltriazolylmethylamine (THPTA), and 0.1 mM biotin picolyl azide), and incubated for 30 min at room temperature, protected from light. The resulting samples were analyzed by sodium dodecyl sulfate-polyacrylamide gel electrophoresis (SDS–PAGE). For HPG-labeled detection, western blotting was performed using streptavidin-HRP (Cell signaling, 3999, 1:5000), and total protein was visualized with SYPRO Ruby staining. FLAG precipitated proteins were identified via anti-FLAG antibody (Proteintech, 66008-3-Ig, 1:2000)

### RT-qPCR

RNA extracted from yeast culture using MasterPure Yeast RNA Purification Kit (Biosearch, MPY03100) was reverse transcribed into complementary DNA (cDNA) using iScript Reverse Transcription Supermix kit (Bio-Rad, 1708841). Quantitative real-time PCR (qPCR) was done by SsoAdvanced Universal SYBR Green Supermix (Bio-Rad, 1725274) with primers found in Supplementary Table S4.

### Liquid chromatography-tandem mass spectrometry (LC-MS/MS)

Two RP containing protein bands (MW: ∼22 and ∼15 kDa) from the 40S fraction and one band (MW: ∼39 kDa) from the 60S fraction were excised from pre-cast polyacrylamide protein gels following visualization with SYPRO-ruby staining. An in-gel digestion was performed with TCEP reduction and IAA alkylation followed by an overnight digestion with either 500 ng of chymotrypsin, 500 ng of ArgC, or 500 ng of GluC for the three respective protein bands. Five hundred nanogram more of enzyme was added the next day for an additional 2-h digest. Peptides were extracted from the gel pieces using 50% acetonitrile and 0.1% trifluoroacetic acid, and then dried down in a vacuum centrifuge. Peptides were resuspended in 20 μl of 2% ACN, 0.1% trifluoroacetic acid for C18 desalting using Millipore ZipTips. Eluted, desalted peptides were dried down in a vacuum centrifuge before being resuspended in 20 μl of 2% acetonitrile, 0.1% formic acid for mass spectrometry (MS) analysis.

A nanoflow ultra high-performance liquid chromatograph (Vanquish Neo) coupled to an electrospray bench top orbitrap mass spectrometer (Orbitrap Ascend Tribrid, Thermo) with FAIMS was used for tandem MS (MS/MS) peptide sequencing experiments. The sample was first loaded onto a pre-column (2 cm × 100 μm ID packed with C18 reversed-phase resin, 5 μm, 100Å) and washed for 8 min with aqueous 2% acetonitrile and 0.04% trifluoroacetic acid. The trapped peptides were eluted onto the analytical column (C18, 75 μm ID × 25 cm, 2 μm, 100 Å, Dionex). The 120 min gradient was programmed as: 95% solvent A (2% acetonitrile + 0.1% formic acid) for 8 min, solvent B (90% acetonitrile + 0.1% formic acid) from 5% to 38.5% in 90 min, then solvent B from 50% to 90% B in 7 min and held at 90% for 5 min, followed by solvent B from 90% to 5% in 1 min and re-equilibrate for 10 min. The flow rate on analytical column was 300 nl/min. Two FAIMS CV values (CV-45 and CV-65) with 1.5 s each cycle time were used to collect tandem mass spectra in a data-dependent manner following each survey scan using 15-s exclusion for previously sampled peptide peaks. MS and MS/MS resolutions were set at 60 000 and 15 000, respectively.

Protein database searching with Proteome Discoverer 3.0 was used to identify peptides using the UniProt *S. cerevisiae* database with Mascot and Sequest. Samples were searched with enzymes set to chymotrypsin, ArgC, and GluC, respectively. Variable modifications included oxidation of methionine, carbamidomethylation of cysteine, and N-terminal acetylation. The precursor mass tolerance was set at 10 ppm and fragment mass tolerance of 0.05 Da, and two missed cleavages were allowed. Scaffold 5.0 and Skyline version 23.1 were used to visualize the data.

### Semi-quantitative proteomic analysis of crude ribosomes by nanoLC-MS/MS

Lyophilized crude 80S ribosomes from WT_rRNA and ES27L *Δb1-4* strain, and WT: W303 and *Δmap1* strain were dissolved in 50 mM ammonium bicarbonate and 6 M urea (for trypsin digestion) or 50 mM Tris–HCl pH 7.5 and 6 M urea (for GluC digestion). Denatured proteins were reduced with 5 mM DTT and alkylated with 50 mM iodoacetamide. Then, the solution was diluted five-fold with 50 mM ammonium bicarbonate or 50 mM Tris–HCl (pH 7.5) to achieve a final area concentration of 1.2 M. 1/20th amount of Trypsin&LysC Mix (Promega, V5073) or GluC protease (Promega, V1651) was added, and incubated overnight at 37°C. The digested peptides were acidified by adding trifluoroacetic acid (at a final concentration of 0.5%) and desalted with GL-tip SDB (GL Sciences, Japan).

The measurement of nanoLC-ESI-MS/MS was performed with a Q-Exactive tandem mass spectrometer and an Easy-nLC1000 nanoflow HPLC system (Thermo Fisher Scientific) in data-dependent acquisition (DDA) mode (Top 10 method). The detailed settings were the same in the previous study [52]. When the analysis for trypsin-digested peptides, +2 to + 4 charged signals were included for MS/MS measurement, and when the analysis for GluC-digested peptides, +2 to + 5 charged signals were included for MS/MS measurement. The measurement was performed three times in each sample as technical replicates. Two biological replicate sets were measured for WT_rRNA and ES27L *Δb1-4* strain, and one set was measured for WT: W303 and *Δmap1* strain.

The obtained data were processed with the Proteome Discoverer (ver. 3.1) bundled with the SEQUEST HT search engine (Thermo Fisher Scientific) for peptide annotation and LFQ quantification. As the dataset for peptide search, the AA sequences of cytosolic ribosomal subunit proteins of *S. cerevisiae* S288c strain from the UniProt database (UP000002311, downloaded on March 27, 2024) were used. As the modifications, Acetylation (+42.010565 Da), Met-loss (–131.040485 Da), and Met-loss & Acetylation (–89.02992 Da) as dynamic modifications at Protein N-terminus, Deamidated (+0.984016 Da) as a dynamic modification on Asp and Glu, Oxidation (+15.994915 Da) as a dynamic modification on Met, and Carbamidomethylation (+57.021464 Da) as a static modification on Cys were defined for peptide search. The intensity was normalized by the total peptide amount. Missing values were imputed by the low abundance resampling method for statistical analyses. For the comparison at a peptide level, the peptides whose FDR confidence calculated by the Percolator method was high (< 0.01) and whose detected number of peptide spectrum matches (PSMs) was >2 were used. For the calculation of fold changes at a protein level, the median of the peptide fold changes of each RP was used since most RPs in *S. cerevisiae* have paralogs with nearly identical AA sequences. The peptides with modifications except carbamidomethyl were omitted for the fold change calculation. The proteins whose detected number of peptides was <3 were excluded from the protein-level comparison. The downstream statistical analyses were performed with the R software (version 4.4.2) with in-house scripts on the Jupyter-notebook platform (version 7.2.2). Violin plots were depicted by using the geom_violin function in the ggplot2 package with default settings.

## Results

### N-terminal processing controls translation fidelity independently of the co-translational process

Different species have developed tailored mechanisms for maintaining the fidelity of mRNA translation, adapted to the average lengths of their proteins and the complexity of their proteomes [4, 7]. Indeed, controlling mRNA translation fidelity through eukaryote-specific rRNA domains has a crucial role in maintaining protein homeostasis [11–13]. We previously discovered that the ES27L enhances translation fidelity by recruiting initiator methionine (iMet) processing enzymes, MetAPs, directly to the ribosomes (Fig. 1A) [11]. In this study, leveraging yeast genetics in combination with an IVT system, we elucidate a novel regulatory mechanism mediated by the RAP, MetAPs.

Since ES27L is the central hub for other RAPs for co-translational processing, we began to look into RAPs involved with co-translational processing. MS analysis of our previous study indicated that the ribosome lacking distal domain of ES27L (ES27L *Δb1-4*) did not reduce the association of NAC or RAC but did reduce association of MetAPs and a subunit of the N-terminal acetyltransferase complex A (NatA) (Supplementary Fig. S1B and Supplementary Table S9) [11]. We further tested the impact of individual NatA subunits on translation fidelity by using deletion strains of catalytic (Δ*ard1*), scaffold (Δ*nat1*), and auxiliary (Δ*nat5*) subunits. To monitor translation fidelity, we use a dual-luciferase gain-of-activity reporter. This reporter encodes Renilla (Rluc) and Firefly luciferase (Fluc) in tandem with a stop codon inserted between them. Then, Fluc activity resulting from the stop codon readthrough was normalized to Rluc activity, then further normalized by Fluc/Rluc from an equivalent “wild-type” construct lacking a stop codon at the middle. None of the NatA mutants showed an increase in stop codon readthrough (Supplementary Fig. S1C). These results show that N-terminal acetylation does not play a significant role in stop codon readthrough errors. Given that MetAPs are the most impacted RAP by the truncation of ES27L, reduced translation fidelity in ES27L *Δb1-4* and *Δmap1* strains is due to the deficiency of iMet processing not from other co-translational steps.

To understand the mechanism through which iMet processing regulates translation fidelity, we examined whether MetAPs/ES27L impacts the fidelity of mRNA translation for MetAP nonsubstrate protein synthesis. Since iMet processing is the first processing step the nascent peptide undergoes while the ribosome continues to translate the remainder of the mRNA, we hypothesized that iMet processing of the nascent peptide only regulates ribosomal decoding of that “ongoing” peptide synthesis. Hence, we expected that iMet processing would only affect MetAP substrate and not nonsubstrate protein synthesis. To test this hypothesis, we created MetAP substrate and nonsubstrate reporters by genetically modulating the second AA, which is the basis of the MetAP substrate selection [53]. We constructed stop codon readthrough reporters by extending the N-terminal by inserting a second AA as alanine (Ala) or lysine (Lys) to create the substrate or nonsubstrate reporter, respectively. The charged Lys residue at the second AA position prevents MetAP from cleaving iMet, thus functioning as a nonsubstrate peptide (Fig. 1B). The UGA stop codon readthrough rate of each reporter was monitored in both the wild-type strain (WT: W303) and deletion strain of the major MetAP (Δ*map1*) in yeast. As a positive control, stop codon readthrough of the substrate reporter increased in the *Δmap1* strain compared to the WT: W303 strain. Unexpectedly, the Δ*map1* strain exhibited increased stop codon readthrough even for nonsubstrate protein synthesis (Fig. 1C). This unexpected result indicates that MetAP controls the translation fidelity of global protein synthesis instead of the “ongoing” iMet processed peptide synthesis. Moreover, this result suggests that iMet processing has a role in translation fidelity independent of co-translational iMet cleavage.

### iMet retention rather than co-translational processing drives translation errors

**Figure 2.**
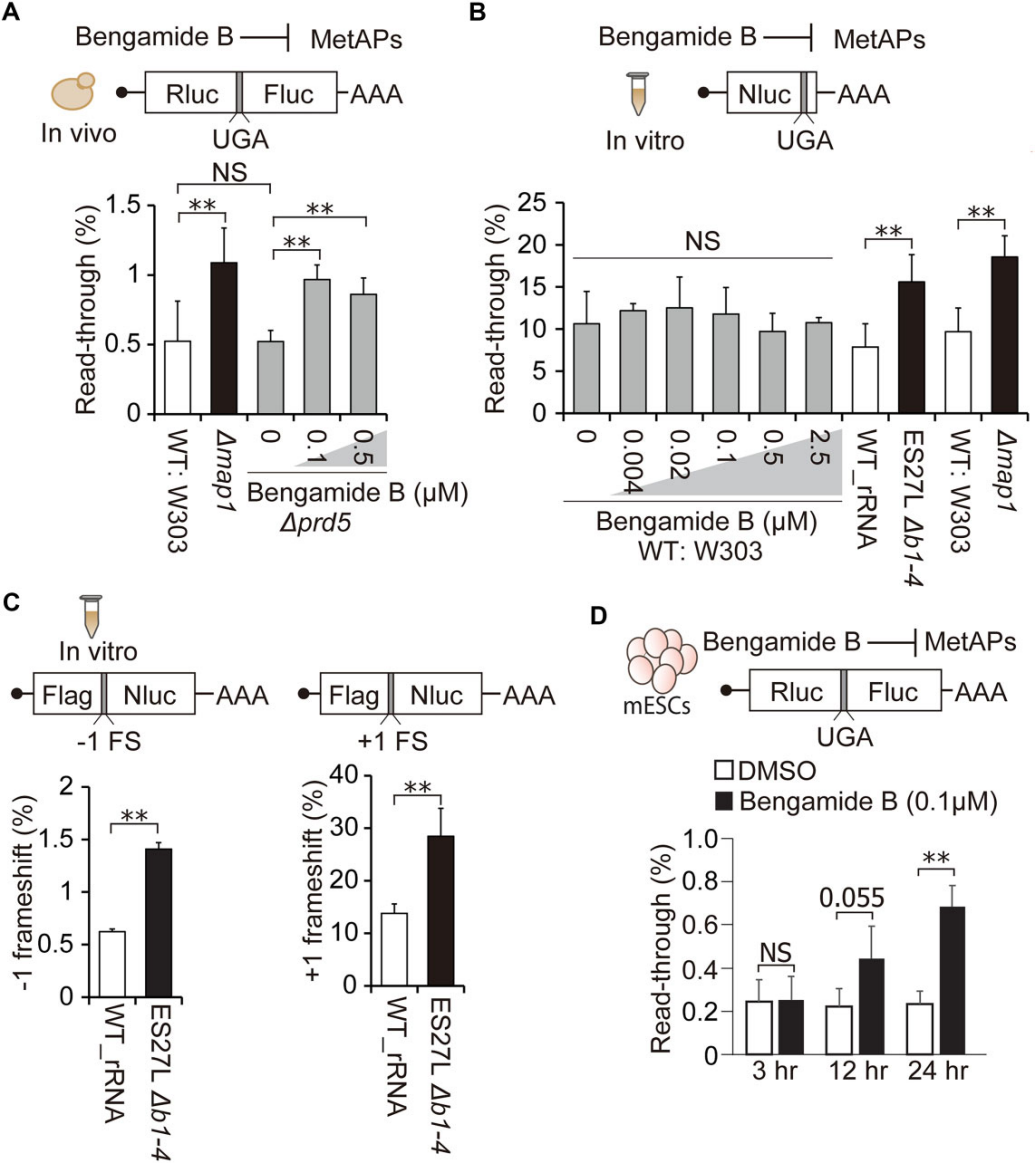
iMet retention rather than co-translational processing drives translation errors. (**A**) Percentage of UGA stop codon readthrough in the presence of the indicated concentration of bengamide B in the *Δpdr5* yeast culture. The WT: W303 and *Δmap1* strains were used as controls, grown in the absence of bengamide B. Data are presented as mean + SD (*t*-test, **P < 0.01; *n* > 3). (**B**) Percentage of UGA stop codon readthrough in the presence of the indicated concentration of bengamide B in the IVT system prepared from the WT: W303 yeast lysate. To measure mistranslation efficiency, Nluc activity from the stop codon construct was normalized to the “wild-type” construct, which does not contain an insertion of the stop codon in the Nluc construct. The IVT systems derived from the WT_rRNA, ES27L *Δb1-4*, WT: W303, and *Δmap1* strains were used as controls, in the absence of bengamide B. Data are presented as mean + SD (*t*-test, **P < 0.01; NS, not significant; *n* > 3). (**C**) Percentage of frameshifts in the IVT systems derived from the WT_rRNA and ES27L *Δb1-4* strains. Frameshift efficiency was measured by normalizing to the “wild-type” construct, which does not contain a frameshift. Data are presented as mean + SD (*t*-test, *P < 0.05; NS, not significant; *n* = 3). (**D**) Monitoring temporal changes of stop codon readthrough after bengamide B treatment in mESCs. Bengamide B was treated at the same time with plasmid transfection. Data are presented as mean + SD (*t*-test, *P < 0.05; NS, not significant; *n* = 3).

Given that Map1 protein impacts the fidelity even for nonsubstrate protein synthesis, one possibility for this effect is that iMet retention on certain cytoplasmic proteins might impact the accuracy of the global translation. To disentangle the effect of co-translational iMet processing from iMet retention on cytoplasmic proteins, we combined an IVT system with the treatment of MetAP inhibitor, bengamide B, which binds to the catalytic pocket of MetAP and inhibits iMet processing [54–56]. We have previously shown that bengamide B treatment increases iMet retention and translation errors in mammalian cells [11]. To apply bengamide B in yeast, we used a strain, *Δprd5*, which has permeabilized cell walls [57, 58]. As expected, the *Δprd5* strain had similar translation fidelity to the WT: W303 strain, and bengamide B treatment increased stop codon readthrough in *S. cerevisiae in vivo* (Fig. 2A). This confirmed the efficacy of bengamide B for inhibiting yeast MetAPs. Utilizing bengamide B in the IVT system enabled us to directly inhibit MetAP and specifically monitor the impact of co-translational iMet processing for the ribosome decoding of “ongoing” peptide synthesis.

To monitor the effect of bengamide B in IVT system, we optimized the translation fidelity reporters for the IVT system. Given that IVT is optimal for shorter protein synthesis, we designed a reporter mRNA with a 171 AA Nano Luciferase (Nluc) instead of the 865 AA long dual-luciferase construct that we used *in vivo*. To monitor stop codon readthrough using Nluc reporter, the histidine (His) at position 88 was substituted with a UGA stop codon so that accurate translation would produce C-terminal truncated Nluc with no activity [59], and stop codon readthrough would produce full-length active Nluc. Nluc activity of the UGA stop codon construct was compared with the WT Nluc construct to calculate stop codon readthrough rate. As an initial validation, we performed IVT in crude WT: W303 cell lysate with different concentrations of paromomycin, which directly binds to the ribosome decoding center and induces stop codon readthrough and frameshifts [60]. Paromomycin increased stop codon readthrough in a dose-dependent manner with our IVT reporters, confirming the capability of quantifying translation fidelity with our IVT system (Supplementary Fig. S2A).

Finally, we assessed the role of co-translational iMet processing by applying bengamide B treatment to the WT: W303 crude IVT system. Surprisingly, the stop codon readthrough rate and the activity of translation in the IVT system remained unchanged under different concentrations of bengamide B (Fig. 2B and Supplementary Fig. S2B), which is a distinct phenotype from *in vivo* bengamide B treatment (Fig. 2A and B). In contrast, IVT lysates produced from ES27L *Δb1-4* and Δ*map1* strains exhibited significantly increased stop codon readthrough compared to respective counterpart wild-type lysate (W303 or WT_rRNA), which was the same phenotype as *in vivo* mutant strains (Fig. 2A and B). This phenotypic discrepancy between *in vivo* and *in vitro* bengamide B treatment is likely due to the lack of cellular accumulation of iMet-retained proteins in the *in vitro* context. This supports our hypothesis that iMet retention on cytoplasmic proteins in mutant strains leads to an increase in translation errors independent of co-translational iMet processing.

To gain a more comprehensive insight into the mutant’s functional impacts on the IVT system, we also used IVT frameshifting reporters, which we described previously [51, 61, 62]. Our reporter contains an insertion of 1 or 2 nt that shifts Nluc out of frame. Therefore, active Nluc is only produced when a frameshift error restores the correct reading frame (Supplementary Fig. S2C). In addition to the stop codon readthrough, the IVT lysate from ES27L *Δb1-4* strain also showed increased ribosome frameshifting in both the +1 and –1 direction compared to cell lysate from the WT_rRNA strain (Fig. 2C), indicating our IVT system from the mutant strain recapitulated the *in vivo* phenotype of decreasing translation fidelity. In addition to the fidelity phenotype, IVT system from *Δmap1* strain also exhibited lower translation activity than IVT from WT: W303 strain, while ES27L *Δb1-4* has similar translation activity to WT_rRNA (Supplementary Fig. S2B). This translation activity difference in IVT system might reflect different growth rate *in vivo*. Indeed, *Δmap1* strain has much slower growth than the WT: W303 strain, in contrast to the similar growth rates between the ES27L *Δb1-4* and WT_rRNA strains [11].

To further test if bengamide B treatment requires some time to accumulate iMet-retained proteins in the cell to increase translation errors, we monitor the temporal change of translation fidelity after bengamide B treatment by taking the advantage of plasmid transfection in cultured cell to compensate for constitutive reporter expression in the yeast system. We treated mouse embryonic stem cells (mESCs) with bengamide B right after reporter plasmid transfection. Then, stop codon readthrough rate at 3, 12, and 24 h after bengamide B treatment were monitored. As we expected, the stop codon readthrough did not increase 3 h after bengamide treatment, which indicates that bengamide B requires some time to accumulate iMet retained proteins to increase stop codon readthrough (Fig. 2D). These results demonstrated that translation error was increased by the accumulation of iMet retained proteins in the cell rather than co-translational iMet processing. Although iMet processing is an essential and abundant process, the functional consequences of iMet retention on specific substrates have not been explored thoroughly. Our findings highlight the specific, yet underappreciated, function of iMet retention on certain proteins that may impact the fidelity of protein synthesis. To further investigate and identify molecular mechanisms underlying translation errors associated with iMet retention, we explored MetAP substrates conserved between *E. coli*, *S. cerevisiae*, and *M. musculus*.

### Ribosomal proteins are conserved substrates of iMet processing

**Figure 3.**
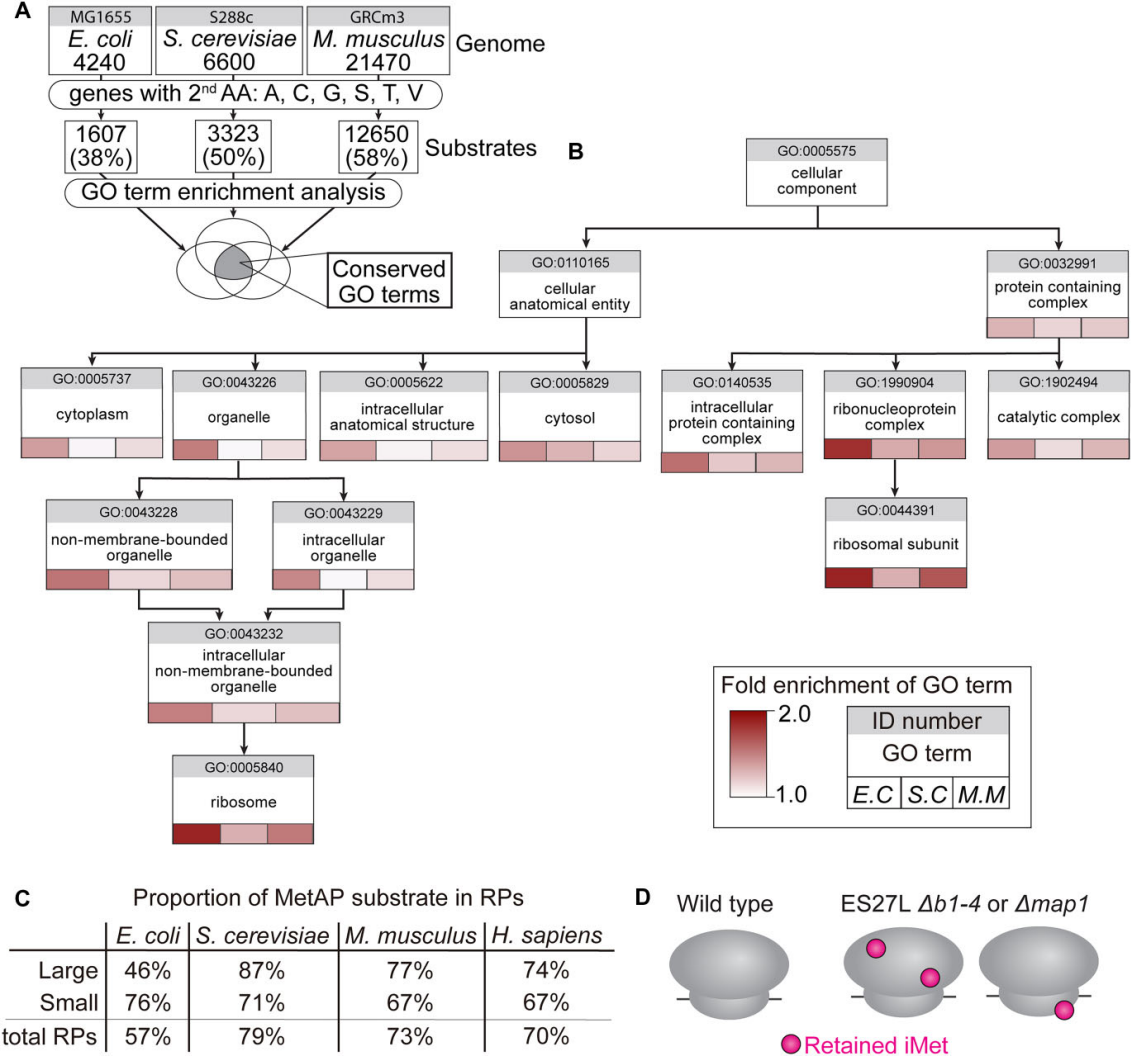
RPs are the conserved substrates of iMet processing. (**A**) Schematic pipeline for GO term enrichment analysis. Each genome from *E. coli*, *S. cerevisiae*, and *M. musculus* was taken for the analysis. Potential MetAP substrates were defined by the small or uncharged second AAs (Ala, Cys, Gly, Ser, Thr, or Val). Overrepresented, enriched GO terms found in all three species are considered conserved GO terms. (**B**) “Ribosome” and “Ribosome subunit” are the most specialized conserved categories in GO term enriched analysis. A hierarchical tree graph of the cellular component category was shown. Only conserved GO terms and one upper layer are shown. Each box represents the GO term and associated ID number. The heat map below the GO term indicates the FDR of the GO term in respective species. GO terms without a heat map indicate a nonconserved term, in at least one species (FDR > 0.05). From top to bottom the terms go from global to specialized categories. Arrow indicated that the child category contains part of genes from parental categories. (**C**) Proportion of cytoplasmic RPs that are MetAP substrates is shown in *E. coli*, *S. cerevisiae*, *M. musculus*, and *H. Sapiens*. The rates of MetAP substrates in small, large, or total RPs were indicated. (**D**) A scheme represented increasing iMet retention on the RPs in the ribosomes in ES27L *Δb1-4* or *Δmap1* strains. Circles indicated iMet retention on RPs.

Despite iMet processing’s critical role, why the complete deletion of MetAPs is lethal and the processing of which MetAP substrates are essential remain unknown. To gain further insight into the function of iMet processing in both cell growth and translation fidelity, we sought to explore the functional characteristics of MetAP substrates genome-wide in multiple species. Given the conservation of MetAP and iMet processing across all domains of life, we hypothesized that there could be conserved iMet substrates critical for cell survival. To test this hypothesis, we conducted a genome-wide GO term analysis of predicted MetAP substrates across three model organisms spanning millions of years of evolution: *E. coli*, *S. cerevisiae*, and *M. musculus*. Potential substrates of iMet processing were defined as annotated open reading frames (ORFs) encoded with small, uncharged second AAs (Ala, Ser, Gly, Cys, Thr, and Val) [53]. Based on this definition, 38% of ORFs in the *E. coli* genome, 50% of ORFs in *S. cerevisiae*, and 58% of ORFs in *M. musculus* were predicted to be MetAP substrates (Fig. 3A). To better understand the biological function of these ORFs, we carried out GO-term enrichment analysis on this list of putative MetAP substrates using PANTHER 19.0 software [49, 50]. The overrepresentation test determined which GO term categories were significantly enriched with putative MetAP substrates (FDR < 0.05) (Supplementary Tables S5–S7). Interestingly, we observed the enrichment of several ribosome-related GO categories/terms, such as “ribosome” and “ribosomal subunit” within the cellular component parent category, “translation” within the biological process category, and “rRNA binding” and “structural constituents of the ribosome” within the molecular function category (Fig. 3B, and Supplementary Fig. S3A and B). These categories were significantly enriched with the list of putative MetAP substrates for all three species. This conserved function of MetAPs for mRNA translation suggests that iMet processing is essential for growth because of a critical role in mRNA translation.

Furthermore, the GO-term “ribosomal subunit” includes many RPs. Interestingly, >70% of all RPs are putative MetAP substrates in *S. cerevisiae* and *M. musculus*, compared to just 57% in *E. coli* (Fig. 3C and Supplementary Table S8). This overall disparity is due to the disproportion of large subunit RPs. Indeed, >70% of eukaryotic large subunit RPs are predicted to be MetAP substrates, in contrast to 45.5% in *E. coli*. The proportion of putative MetAP substrates within small subunit RPs in *E. coli* is similar to that of *S. cerevisiae* and *M. musculus* (Fig. 3C and Supplementary Table S8). Based on the enrichment as putative MetAP substrate in RPs across species, we hypothesize that ES27L *Δb1-4* and Δ*map1* strains increase iMet retention on RPs (Fig. 3D). We propose that iMet retention in RPs might distort the tightly packed structure of the ribosome and affect its translation fidelity in mutant ribosomes.

### Defect in iMet processing does not affect to the composition of RPs in the ribosome

**Figure 4.**
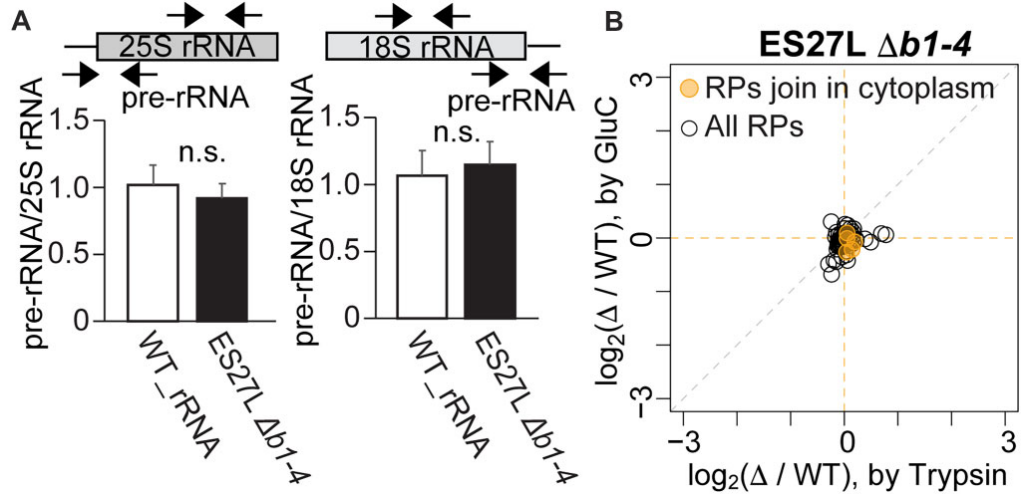
Ribosomes in ES27L Δb1-4 strain do not have delay in ribosome maturation and do not lack RPs. (**A**) ES27L *Δb1-4* strain did not increase premature rRNAs. Pre-rRNA in both 25S and 18S rRNAs was monitored in ES27L *Δb1-4* strain by RT-qPCR. (**B**) Composition of RPs in the ribosomes did not changes between WT_rRNA and ES27L Δ*b1-4* strains. The median of the fold change values for each RP was depicted in the graph. Late joining RPs are highlighted in orange. Fold change values and corresponding *P*-values adjusted by the Benjamini–Hochberg method are listed in Supplementary Dataset S13.

Although we hypothesized that iMet retained RPs will be incorporated into the ribosomes, another possibility is that iMet retention on the RPs affects the stability of RPs. Since the protein N-terminal has a crucial role in protein stability through the N-end rule [63–66], iMet processing may impact the protein half-life. Indeed, N-terminal methionine can be the target of the Arg/N-end rule in the case of bulky charged second AA, which are not substrates of MetAPs. However, there is no existing research testing if iMet retention on expected MetAP substrates become a target of the N-end rule. If the stability of RPs is changed, it might affect the maturation of ribosomes and final composition of incorporated RPs, which may further affect translation fidelity. First, we monitored the amount of mature and premature ribosomes using qPCR detecting pre-rRNAs in both 18S and 25S. The ratio of pre/total rRNA did not change between WT_rRNA and ES27L *Δb1-4* strains (Fig. 4A). We further compared amount of each RP by a semi-quantitative proteomic approach using nanoLC-MS/MS for crude ribosomes from WT_rRNA and ES27L *Δb1-4* strains, which quantified 74 RPs by Trypsin and 66 RPs by GluC treatments. Consistent with RT-qPCR, we did not observe consistent changes across replicates and treatment even for the RPs joining the ribosome in the cytoplasm (Fig. 4B and Supplementary Table S13). Although this result could not exclude the possibility that small amounts of heterogenous ribosomes produce translation errors, it does indicate that ES27L *Δb1-4* strain does not have a defect in ribosome maturation.

### Novel method for monitoring iMet retention

**Figure 5.**
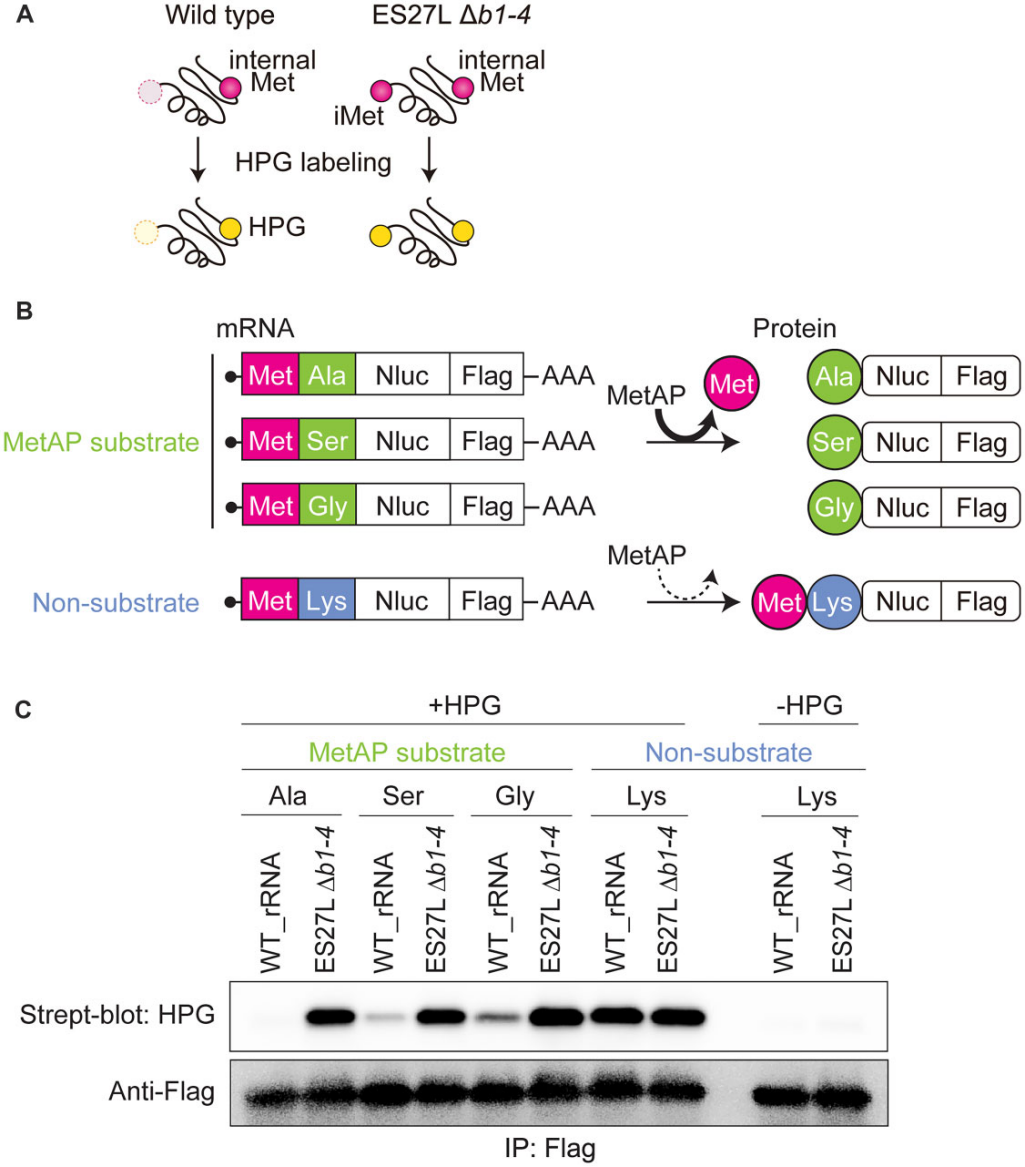
The novel method enables to monitoring of iMet processing *in vivo* using clickable methionine analog. (**A**) Scheme represents the working model that labeling with methionine analog, HPG, enables us to monitor iMet retention in ES27L *Δb1-4* strain. HPG can be incorporated in both initiator methionine (iMet) and internal Met. (**B**) Schematic of the reporter constructs used for proof-of-concept experiment to monitor iMet processing. Each reporter construct has different second AAs representing MetAPs substrate (alanine, serine, or glycine) or nonsubstrate (lysine) followed by Nluc-Flag. (**C**) HPG labeling can monitor the iMet processing of MetAP substrates and nonsubstrate constructs in WR_rRNA and ES27L *Δb1-4* yeast strains. Streptavidin-HRP blotting (Strept-blot) indicated HPG signal, representative of iMet retention, in the reporter derived proteins. Flag western blotting (Anti-Flag) was shown as a loading control. Nluc-Flag reporter protein was purified by Flag immunoprecipitation (IP) from WT_rRNA or ES27L *Δb1-4* lysate cultured with or without HPG.

To test our hypothesis that iMet retention on ribosomes is responsible to the translation fidelity changes in mutant strains, we sought to monitor iMet processing. Effective monitoring of iMet processing remains a challenge. There is currently no method for selectively enriching or detecting peptides or proteins that retained iMet. Therefore, to overcome this challenge, we developed a novel method for detecting iMet retention. Methionine has historically been a labeling target for *in vivo* quantification of translational activity by radioactive S-35 methionine or nonradioactive methionine analogs such as HPG [67, 68]. HPG is a nontoxic, clickable analog of methionine, and yeast can incorporate HPG into newly synthesized proteins at the site of methionine [69]. We thought to apply this methionine labeling technique to monitor iMet processing through differences in HPG signals between WT and mutant strains (Fig. 5A). Since MetAPs can process HPG as methionine *in vitro* [70, 71], HPG retained at the N-terminal can be biotinylated by click chemistry and visualized by blotting using streptavidin-HRP.

As a proof-of-concept experiment to monitor iMet processing with HPG labeling, we prepared Nluc constructs that were MetAP substrates (Met-Ala-, Met-Ser-, and Met-Gly-) and non-MetAP substrate (Met-Lys-). Internal methionines in Nluc constructs were removed by point mutations, and a C-terminal Flag peptide was added for subsequent purification (Fig. 5B). The HPG processing of these reporter constructs was monitored in WT_rRNA and ES27L *Δb1-4* strains. More than expected, the ES27L *Δb1-4* strain tremendously reduced MetAP activity and retained HPG in all MetAP substrate constructs at a similar level to the nonsubstrate construct (Fig. 5C). It is surprising that the loss of ribosome association of MetAP provides such a profound change to N-terminal processing of cytoplasmic proteins. We also noticed that the efficiency of iMet removal in the WT_rRNA strain was different between MetAP substrate constructs depending on the identity of the second AA (Ala > Ser > Gly), thus replicating previous observations and highlighting the sensitivity of this assay (Fig. 5C) [72]. In contrast, the nonsubstrate (Met-Lys-) constructs retained HPG in both WT_rRNA and ES27L *Δb1-4* strains (Fig. 5C). Therefore, these results demonstrated that HPG labeling can effectively monitor differential iMet processing, thus establishing a powerful tool with wide applications. With this method in place, we sought to detect iMet retention on ribosomes especially RPs in mutant strains compared to WT strains.

### iMet retention on the RPs increases in mutant ribosomes

Leveraging our novel method to monitor iMet processing, our goal was to reveal how iMet retention influences the accuracy of translation. To this end, since RPs are conserved MetAP substrates, we aimed to assess our hypothesis that mutant (ES27L *Δb1-4* and Δ*map1*) strains increase iMet retention on RPs in the ribosomes. Given that HPG will be incorporated in both initiator and internal methionines, detecting iMet retention with HPG will be more challenging with samples that have more internal methionine or complex proteome. However, methionine is the overall second rarest AA, amounting to only 2% of the proteome [73, 74]. Indeed, 67 of 79 RPs in *S. cerevisiae* have less than three methionine residues, including iMet. Thus, our novel iMet processing assay might be able to monitor the iMet retaining proteins in the purified ribosomes and detect differences in iMet retention between strains.

**Figure 6.**
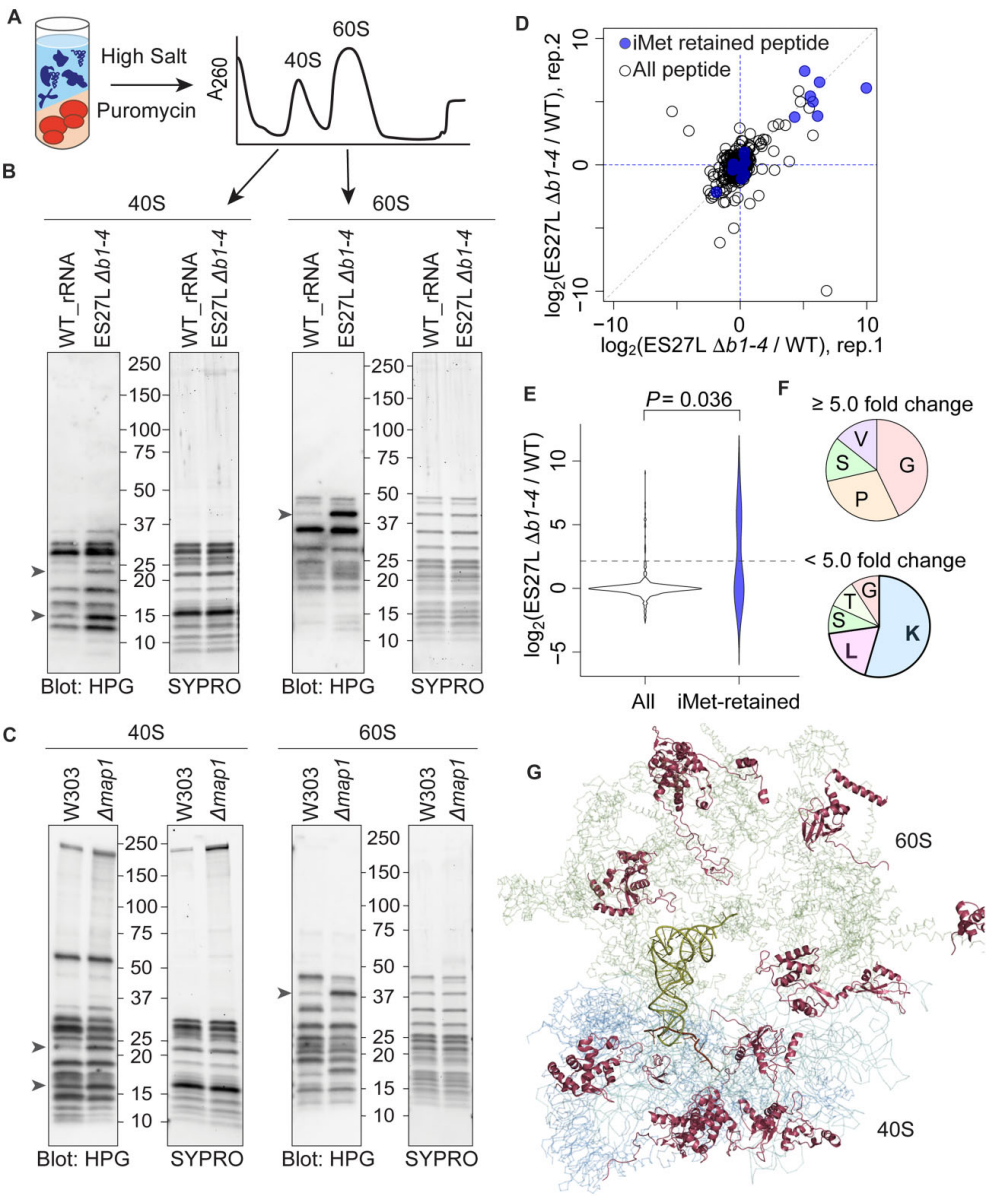
Ribosomes in ES27L *Δb1-4* and *Δmap1* strain increase iMet retaining RPs. (**A**) The scheme of the experimental procedure indicates puromycin treatment of crude 80S ribosome under high salt conditions separates the 40S small subunit and 60S large subunit, which can be fractionated by sucrose density gradient. (**B**) Pure 40S & 60S ribosomal subunits indicated more RPs that increase HPG signals in ES27L *Δb1-4* ribosomes. RPs identified by LC-MS/MS indicated by arrows. (**C**) Pure 40S & 60S ribosomal subunits from WT: W303 and *Δmap1* strains indicated that consistent RPs increase HPG signal in ES27L*Δb1-4* and *Δmap1*. (**D**) A scatter plot shows fold changes of RP peptides between WT_rRNA and ES27L *Δb1-4* strains determined by the LC-MS/MS analysis. The fold changes for two biological replicates at a peptide level were compared. Fold change values and corresponding *P*-values adjusted by the Benjamini–Hochberg method were listed in Supplementary Dataset S11. (**E**) iMet retained peptides increased in ES27L *Δb1-4* strain. Distribution of the fold change of the peptides between WT_rRNA and ES27L D*b1-4* strains were shown as violin plots. The numbers of all peptides and iMet-retained peptides were 768 and 18, respectively. The *P*-value was obtained by the Wilcoxon rank sum test (two-sided). The dashed horizontal line indicates *y* = log_2_(5). (**F**) The iMet-retained peptides increased >5-fold in ES27L *Δb1-4* strain enriched with small and noncharged second AAs. Seven peptides were detected for the ≥5-fold change group and 11 peptides were detected for the <5-fold change group. (**G**) LC-MS/MS identified iMet retained RPs (cartoon) were visualized using Pymol in overall yeast ribosome structure (ribbon) (PDB: 4v6i).

Therefore, we first fractionated cell lysate of the crude ribosomes and cytosolic fractions from HPG-labeled WT_rRNA and ES27L *Δb1-4* strains utilizing a sucrose cushion (Supplementary Fig. S4A). Although there are no distinct HPG signal discrepancies in the input lysate and cytoplasmic fraction (Supplementary Fig. S4B and C), we observed a single band in the ribosome fraction that increased intensity in ES27L *Δb1-4* compared to WT_rRNA ribosomes (Supplementary Fig. S4D). However, the crude ribosome contains a wide range of proteins, including RAPs and both small 40S and large 60S subunit proteins. For further purification, crude ribosomes were washed with high salt to remove RAPs, and 40S and 60S subunits were separated by puromycin treatment. Then, each 40S and 60S subunits were isolated by sucrose density gradient (Fig. 6A). The separation of subunits makes it easier to observe the signal differences between WT_rRNA and ES27L *Δb1-4* ribosomes. Remarkably, three bands repeatedly exhibited stronger signals in the ES27L *Δb1-4* ribosome (Fig. 6B). A 39 kDa protein band consistently showed a difference in crude ribosomes and the 60S subunit. Two additional bands with distinct HPG intensities were observed in the purified 40S subunit. These three bands were also discerned in the isolated subunits from WT: W303 and Δ*map1* strain ribosomes (Fig. 6C). By performing LC-MS/MS on three distinct bands, we identified 60S 39 kDa band as uL4, 40S 22 kDa band as uS4/eS7, and 40S 15 kDa band as uS11. Importantly, we were able to detect iMet retaining N-terminal peptides from uL4 and uS11 in ES27L *Δb1-4* samples but not from WT_rRNA samples (Supplementary Fig. S5A and B). Although these bands were particularly sensitive to disruptions of MetAP recruitment by ES27L, there could be more iMet retained RPs hidden by the strong background HPG signals.

Aiming to further identify the iMet retaining RPs, we deeply analyzed our semi-quantitative proteomic nanoLC-MS/MS data by focusing on the N-terminal peptides. Strikingly, iMet retained peptides exhibited larger differences between mutant and WT than other peptides (Fig. 6D and E, and Supplementary Table S11). There are two distinct populations in the iMet retained peptides detected by LC-MS/MS. Interestingly, iMet peptides, which were increased in ES27L *Δb1-4* strain, tend to have small uncharged second AA, Gly, Pro, Ser, and Val. In contrast, iMet peptides, which did not change between strains, were enriched with charged or large second AA, Lys and Leu, which are not the MetAP substrate (Fig. 6F). We also performed LC-MS/MS analysis for *Δmap1* and WT: W303 ribosomes and observed a similar trend in iMet retained peptides (Supplementary Fig. S6A–C and Supplementary Table S12). In total, we detected 14 RPs increased iMet retained peptides in ES27L *Δb1-4* or *Δmap1*, including 9 small subunit and 5 large subunit RPs (Fig. 6G and Supplementary Table S10). These include uL4 and uS4, which are the RPs identified in Fig. 6B and C. Although the tendency was not strong (∼4-fold change against wild-type), eS7 also seems to increase iMet retention. As a whole, these data demonstrated that mutant ribosomes increase iMet retention on RPs resulting from decreased MetAP activity. Interestingly, we observed significant decline of eL24 in the protein level only in *Δmap1* but not in ES27L *Δb1-4* strain (Supplementary Fig. S6D and Supplementary Table S14). eL24 is late joining nonessential RP. Strain with complete deletion of eL24 has slow growth phenotype with paromomycin sensitivity [75], similar to *Δmap1* strain. Given that eL24 has critical function for the translation elongation, it is possible that lacking eL24 in *Δmap1* strain reduces translation fidelity. To further characterize the functional consequence of these changes in the ribosomes, we purified iMet retaining ribosomes from ES27L *Δb1-4* or *Δmap1* strain, then monitored translation activity and fidelity of purified ribosomes using our IVT system.

### iMet retaining ribosome is error-prone

**Figure 7.**
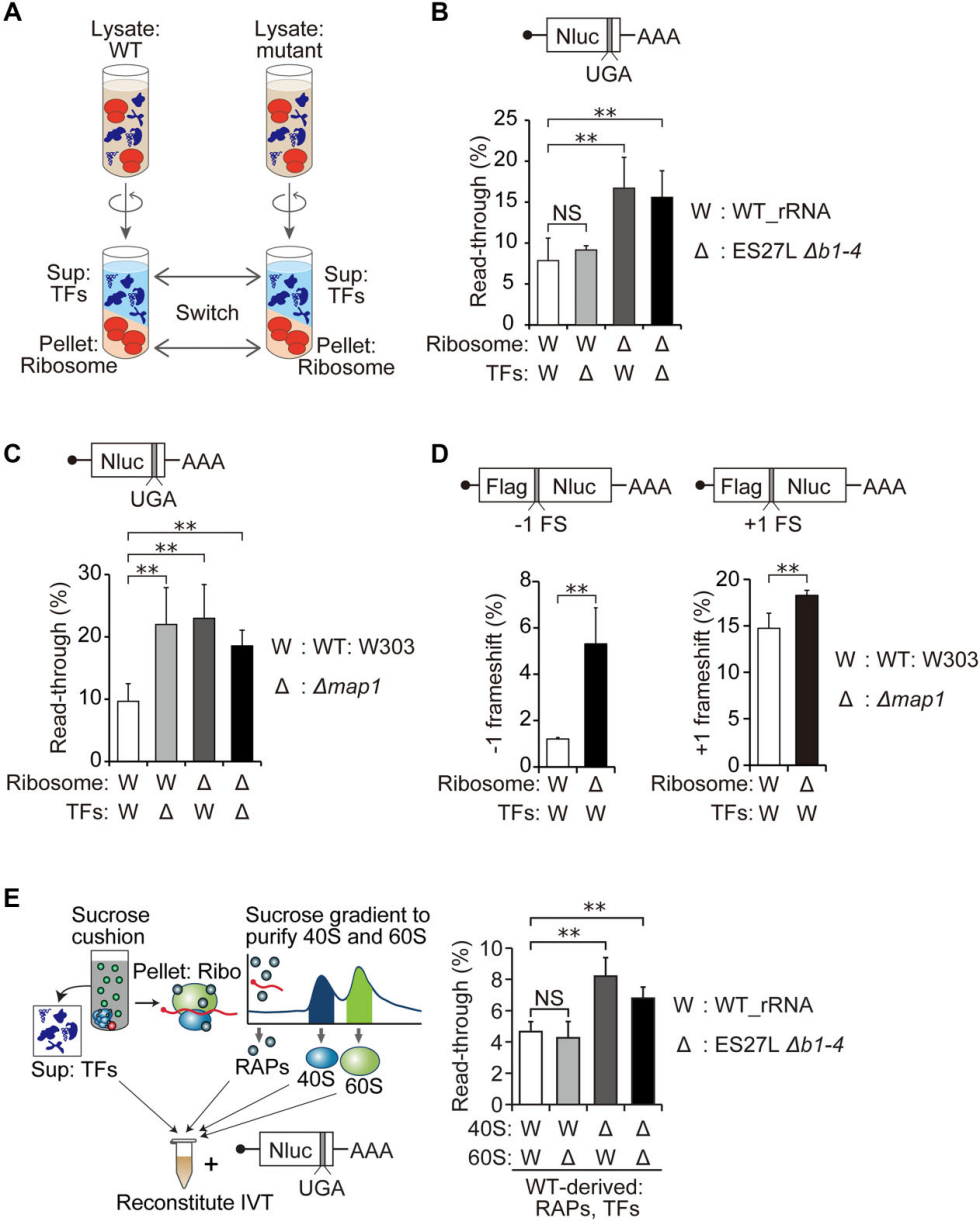
Reconstitution of IVT system with purified ribosomes demonstrated that ribosomes in ES27L *Δb1-4* and *Δmap1* are error-prone. (**A**) Schematic of the ribosome-switching IVT system. After ultracentrifugation of wild-type and mutant yeast lysates, TFs from the supernatant and ribosomes from the pellet were prepared. The IVT systems were reconstituted with different combinations of TFs and ribosome fractions exchanged between strains. (**B**) Percentage of UGA stop codon readthrough in the IVT systems reconstituted with all combinations of TFs and ribosomes between the WT_rRNA (W) and ES27L *Δb1-4* (Δ) strains. Data are presented as mean + SD (*t*-test, **P < 0.01; NS, not significant; *n* > 3). (**C**) Percentage of UGA stop codon readthrough in the IVT reconstituted from purified ribosomes and TFs from the WT: W303 (W) and *Δmap1* (Δ) strains. Data are presented as mean + SD (*t*-test, **P < 0.01; *n* > 3). (**D**) Percentage of frameshifts in both -1 and + 1 directions increased with ribosomes from the *Δmap1* (Δ) strain in the reconstituted IVT systems. Data are presented as mean + SD (*t*-test, *P < 0.05; *n* = 3). (**E**) (Left) Schematic of the purification process for TFs, RAPs, 40S, and 60S for IVT reconstitution. Ribosomes were treated with high salt and puromycin to split the subunits, followed by ultracentrifugation through a sucrose density gradient to fractionate 40S, 60S, and RAPs. These components were recombined with TFs to reconstitute the IVT system. (Right) Percentage of UGA stop codon readthrough in IVT systems reconstituted with purified ribosomal subunits from WT_rRNA (W) and ES27L *Δb1-4* (Δ) strains. The 40S subunit from ES27L *Δb1-4* strain increases translation errors. Data are presented as mean ± SD (two-tailed *t*-test, **P < 0.01; NS, not significant; *n* > 3).

Leveraging our IVT system which recapitulates the *in vivo* translation fidelity of ES27L *Δb1-4* and *Δmap1* in crude IVT lysates (Fig. 2B and C), we examined the functional impact of iMet retention on the ribosome by fractionating crude IVT lysate from WT_rRNA and ES27L *Δb1-4* strains into TFs and ribosomes. By testing all combinations of the purified ribosomes and TFs of WT_rRNA and ES27L *Δb1-4*, we assessed the impact of iMet retention from each fraction on the translation activity and fidelity (Fig. 7A). Each isolated TF or ribosome fraction lacked translation activity on their own, indicating minimal cross-contamination between the two (Supplementary Fig. S7A). When we combined WT ribosomes and WT TFs, we were able to reconstitute IVT activity (Supplementary Fig. S7A). Using this system, we first tested the translation activity of each combination using the WT Nluc construct mRNA and observed similar translation activity in all combinations (Supplementary Fig. S7B).

Utilizing our ribosome reconstitution protocol with UGA stop codon readthrough reporter for IVT, we next examined the fidelity of mRNA translation in all combinations of ribosomes and TFs fractions between WT_rRNA and ES27L *Δb1-4* strains. We observed that the ES27L *Δb1-4* ribosomes (that have lost the docking site for MetAP and thus likely retain iMet) with either TFs fractions from WT or ES27L *Δb1-4* strains increase stop codon readthrough, indicating that iMet retention on ribosomes results in ribosomes being error-prone (Fig. 7B). Although the ribosome fraction contains RAPs and small amounts of TFs, the TFs fraction contains significantly more of these proteins. The combination of WT ribosomes with the TFs fraction from ES27L *Δb1-4* strain did not increase translation errors (Fig. 7B), indicating that TFs and RAPs from ES27L *Δb1-4* strain do not contain any iMet retaining proteins that increase translation errors. These results demonstrate that iMet retention on the RPs in the ribosomes in ES27L *Δb1-4* strains increased translation errors without affecting translation activity.

In addition to the ES27L *Δb1-4* strain, IVT reconstitution of purified Δ*map1* ribosomes and TFs was performed. Interestingly, we observed a more substantial reduction in translation activity from the *Δmap1* TFs than from *Δmap1* ribosomes (Supplementary Fig. S7C). In contrast to the ES27L *Δb1-4*, both TFs and ribosome fraction from *Δmap1* strain increase UGA stop codon readthrough (Fig. 7C). The ribosomes from *Δmap1* strain also increase ribosomal frameshifting (Fig. 7D). Importantly, these results indicate that supplementation of Map1 protein in the IVT from WT TFs fraction could not rescue translation fidelity (Fig. 7C and D).

### iMet retention(s) on the 40S subunit increase translation errors

To identify the causative ribosomal subunit, we further isolate each ribosomal subunit and RAPs using a sucrose density gradient. Then, IVT was reconstituted from purified 40S and 60S, as well as TFs and RAPs (Fig. 7E). Our reconstituted IVT is still sensitive to paromomycin in a dose-dependent manner (Supplementary Fig. S7D). Since most of the RAPs also exist in the TFs fraction, RAPs in the top fraction of the sucrose gradient are not essential but increased translation activity with reconstitute IVT (Supplementary Fig. S7E). Here, we focused on purified 40S and 60S subunits from both WT_rRNA and ES27L *Δb1-4* strains. Strikingly, only when the 40S subunit from ES27L *Δb1-4* was used for reconstitution, we observed an increasing stop codon readthrough without significant changes in translation activity (Fig. 7E and Supplementary Fig. S7F). Given that ES27L exists in the 60S subunit and the 60S subunit from ES27L *Δb1-4* strain did not increase stop codon readthrough errors, these results clearly distinguish the function of ES27L and the observed translation fidelity phenotype. This demonstrates that iMet retention(s) on small subunit RPs is the causative factor behind increased translation errors. Overall, our findings elucidated the molecular basis of translation fidelity regulation through eukaryote-specific rRNA domain, ES27L, by fine-tuning MetAPs activity of iMet processing for specific RPs.

## Discussion

This research elucidates the molecular mechanism of translation fidelity control through the extension of a single eukaryote-specific rRNA domain, ES27L, and its recruitment of the highly conserved RAP, MetAP. We demonstrated that MetAP not only impacts the translation fidelity of MetAP substrate protein synthesis but also extends its impact to the fidelity of nonsubstrate protein synthesis. To unravel how ribosomal association of MetAP contributes to the fidelity of global protein synthesis, we leveraged an IVT system to separate the effect of accumulated iMet retention from co-translational iMet processing and the role of ES27L in the 60S subunit. Instead, we identified that 40S ribosomal subunit from ES27L *Δb1-4* strain is the causative factor that induces translation errors. By developing a novel method to monitor iMet retention using a nonradioactive, clickable methionine analog, we demonstrated that ribosomes in ES27L *Δb1-4* and *Δmap1* strains selectively increased iMet retention on RPs. Therefore, we propose a model in which ES27L recruits MetAP to the ribosome to ensure proper iMet processing of nascent proteins. In the absence of this recruitment, iMet-retained nascent proteins—including RPs—accumulate and are incorporated into ribosomes, where they distort the tightly packed ribosomal structure and induce translation errors. These findings highlight a novel mechanism by which the eukaryotic ribosome contributes to its own structural integrity and translational accuracy by spatially regulating the activity of universally conserved MetAP enzyme through eukaryote-specific ES27L.

Eukaryotic ribosomes have a more packed structure than bacterial ribosomes, with extended rRNA by ESs and having more RPs [9]. Therefore, even small changes can have significant impacts on the ribosome structure and can affect the functionality of the ribosomes. Indeed, just three AA C-terminal extensions in eS24 under hypoxia can provide significant changes in mRNA translation regulation [76]. There are multiple RP paralogs where differences in only a few AA can impact translation regulation, such as uL30 A and B [77]. Although the ribosomes in both ES27L *Δb1-4* and *Δmap1* strains are affected and increase iMet retained RPs, ribosomes between these two strains have some differences. Indeed, ES27L *Δb1-4* strain partially inhibits both Map1 and Map2 activity, in contrast, *Δmap1* strain has standard Map2 activity with no Map1 activity. Given that the Map1 and Map2 have different substrate specificity, the impact on the ribosome will be different. Indeed, eL24 is only reduced in *Δmap1* strain but not in ES27L *Δb1-4* strain. Interestingly, MetAP1 and MetAP2 (Map1 and Map2 in yeast) have different tissue expression patterns [78, 79]. As a result, different tissues and cell types, including cancer cells [80, 81], may exhibit distinct MetAP activity and varying levels of iMet processing on RPs. Notably, cancer cells often have sequence variations in ES27L [18], which might fine-tune the MetAP activity in cancer cells.

Our immediate follow-up question is which specific RP or combination of RPs with iMet retention impacts the translation fidelity. Our IVT system with exchanging ribosomes and subunits between strains demonstrated that iMet retention on the RPs in 40S subunit causes error-prone ribosomes in ES27L *Δb1-4*. We identified a total of nine small subunit RPs that increased iMet retention by LC-MS/MS from the gel and crude ribosomes (eS7, eS17, eS19, eS24, eS27, uS3, uS4, uS11, and uS12). Mutations in eS7, eS17, eS19, eS24, and eS27 have been associated with Diamond-Blackfan anemia (DBA) [82]. The uS4, uS11, and uS12 are known to be important for translation fidelity [7, 83, 84]. Therefore, iMet retention on these RPs might affect the fidelity of ribosomal function and increase translation errors. Our finding emphasizes that the ribosome is a precisely assembled complex structure; even a single AA change, such as iMet retention, can distort the structure and interfere with its function. This principle can be applied to the other complexes. Indeed, GO term analysis on predicted MetAP substrates shows enriched “protein containing complex” category, which includes the proteasome and DNA and RNA polymerases. Given that the mitochondria and chloroplast also have a critical protein complex—ATP synthase—our study not only elucidates the molecular mechanism by which iMet processing influences translation fidelity but also proposes the broader molecular principles governing the importance of iMet processing in maintaining cellular function.

Given the critical role of N-terminal for protein half-life, even though we did not observe the differences in the composition of RPs in the ribosome in the ES27L Δ*b1-4* strain (Fig. 4B), it is possible that these strains have an impact on protein half-lifes, including free RPs. Indeed, ES27L *Δb1-4* strain can affect N-terminal acetylation (Fig. 1A). N-terminal acetylation of methionine and other AAs create an Ac/N-degron that can be recognized for ubiquitination [85]. Given that N-terminal degrons could be more accessible when proteins are not in the protein complexes [86], defect in N-terminal processing of RPs might have a role in the clearance of free RPs to eliminate excess amounts of RPs [87]. Especially in mammalian cells, free RPs trigger the p53 pathway [88]. Therefore, N-terminal processing through ES27L might contribute to various biological processes not only translation fidelity.

## Supplementary Material

gkaf448_Supplemental_Files

## Data Availability

The data underlying this article are available in the article and in its online Supplementary Data.

## Notes

Present address: Department of Molecular, Cellular, and Developmental Biology, Yale University, New Haven, CT 06511, United States
