# Supplementary material for: Ribosomal expansion segment contributes to translation fidelity via N-terminal processing of ribosomal proteins
Source: Nucleic Acids Res. 2025 May 28;53(10):gkaf448. doi: 10.1093/nar/gkaf448 (PMC12117404; doi:10.1093/nar/gkaf448)
Supplement: gkaf448_Supplemental_Files [file gkaf448_supplemental_files.zip › Supplemental Information Rev2.pdf]

Supplemental Information

Ribosomal Expansion Segment Contributes to Translation Fidelity via N-terminal Processing of Ribosomal Proteins

Riku Nagai, Olivia L Milam, Tatsuya Niwa, William J Howell, Jacob A Best, Hideji Yoshida, Carver D Freeburg, John M Koomen, and Kotaro Fujii

Including

Supplementary Figure S1 to S7

Supplementary Table S1 to S14

Figure S1

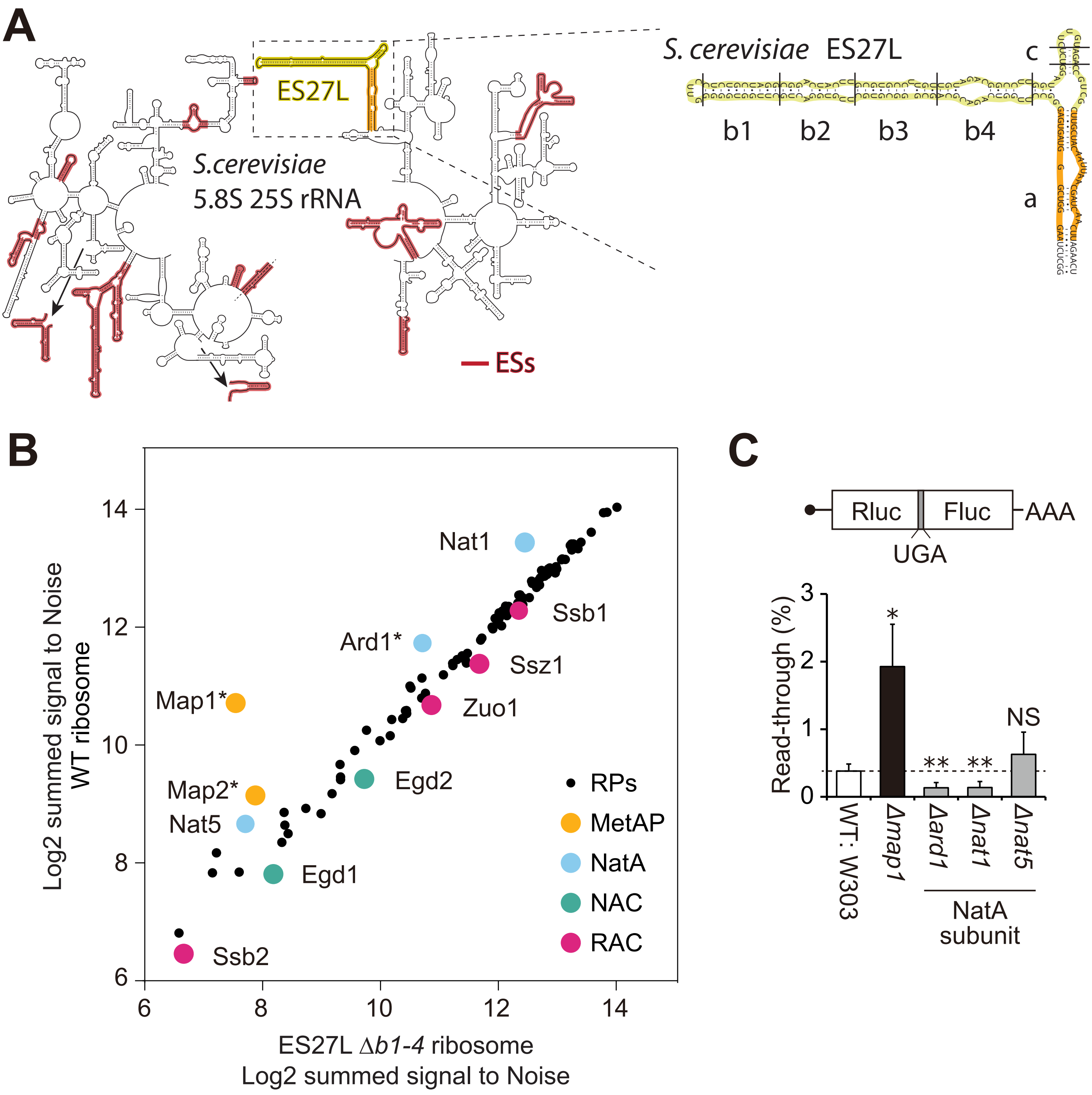
A
ES27L
S.cerevisiae
5.8S 25S rRNA
ESs
S. cerevisiae ES27L
b1
b2
b3
b4
a
c
B
Nat1
Ssb1
Ard1*
Ssz1
Map1*
Zuo1
Egd2
Map2*
Nat5
Egd1
Ssb2
RPs
MetAP
NatA
NAC
RAC
Log2 summed signal to Noise
WT ribosome
ES27L Δb1-4 ribosome
Log2 summed signal to Noise
C
Rluc
Fluc
AAA
UGA
Read-through (%)
*
**
**
NS
WT: W303
Δmap1
Δard1
Δnat1
Δnat5
NatA
subunit

Figure S1. Translation fidelity regulation through ES27L is independent of other ribosome associated proteins (RAPs) for co-translational processing.

(A) Secondary structure of 25S and 5.8S rRNA in *S.cerevisiae*. ES27L Δb1-4 strain has deleted ES27L b1 to b4. ESs: expansion segments. **(B)** Data re-mining of tandem mass tag (TMT) mass spectrometry (MS) indicates the changes and non-changes between the WT_rRNA and ES27L Δ*b1-4* ribosomes in RAPs including MetAPs (yellow), N-Acetyl transferase (NatA) complex (blue), nascent-polypeptide-associated complex (NAC) (green), and ribosome associated complex (RAC) (red). The ribosomal proteins (RPs) (black) are highly consistent. The data set of TMT-MS was obtained from elsewhere (Supplementary table S9). *FDR<0.05 **(C)** Percentage of UGA stop codon readthrough was monitored in the NatA subunit-deletion strains. To measure stop codon readthrough rate, normalized Fluc activities relative to Rluc were further normalized to the "wild-type" construct, which does not have an insertion of the stop codon between Rluc and Fluc. The WT: W303 and *Δmap1* strains were used as controls. Data are presented as mean + SD (t-test, **$p < 0.01$; $n > 3$).

Figure S2

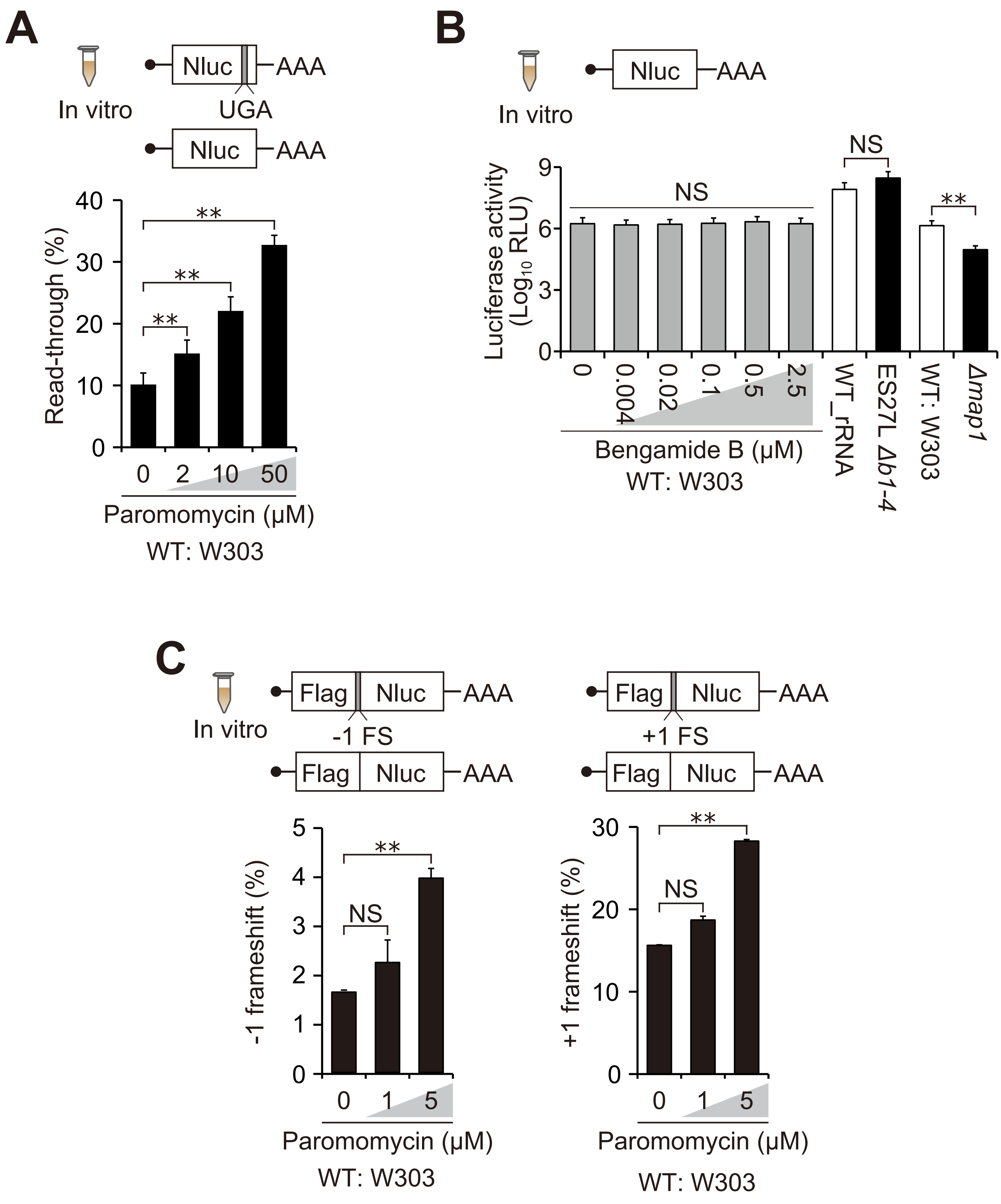
A
In vitro
Nluc
UGA
AAA
Nluc
AAA
Read-through (%)
40
30
20
10
0
**
**
**
0
2
10
50
Paromomycin (μM)
WT: W303
B
In vitro
Nluc
AAA
Luciferase activity
(Log10 RLU)
9
6
3
0
NS
NS
**
0
0.004
0.02
0.1
0.5
2.5
Bengamide B (μM)
WT: W303
WT_rRNA
ES27L Δb1-4
WT: W303
Δmap1
C
In vitro
Flag
Nluc
AAA
-1 FS
Flag
Nluc
AAA
Flag
Nluc
AAA
+1 FS
Flag
Nluc
AAA
-1 frameshift (%)
5
4
3
2
1
0
**
NS
0
1
5
Paromomycin (μM)
WT: W303
+1 frameshift (%)
30
20
10
0
**
NS
0
1
5
Paromomycin (μM)
WT: W303

Figure S2. Establishing in vitro translation (IVT) system to monitor translation fidelity.

(A) Dose-dependent translation errors induced by paromomycin in the crude IVT lysate produced from the WT: W303 strain. Percentage of UGA stop codon readthrough was monitored by the UGA containing Nluc construct normalized by the "wild-type" Nluc construct without stop codon in the coding sequence (CDS), which indicates the translation activity. Data are presented as mean + SD (t test, $^{**}p < 0.01$; $n > 3$). **(B)** Translation activity of the IVT system in the presence of the indicated concentration of bengamide B. IVT lysate was prepared from the WT: W303 yeast. To measure translation activity, activity from the "wild-type" Nluc construct was monitored. The IVT systems derived from the WT_rRNA, ES27L *Δb1-4*, WT: W303, and *Δmap1* strains were used as controls, in the absence of bengamide B. Data are presented as mean + SD (t-test, $^{**}p < 0.01$; NS, not significant; $n > 3$). **(C)** Dose-dependent -1 and +1 ribosomal frameshift errors induced by paromomycin in the crude IVT lysate produced from the WT: W303 strain. Percentage of frameshift was monitored by the -1/+1 frameshift Nluc construct normalized by the "wild-type" Nluc construct without frameshift in the CDS. Data are presented as mean + SD (t test, $^{**}p < 0.01$; $^{*}p < 0.05$; NS, not significant; $n = 3$).

Figure S3

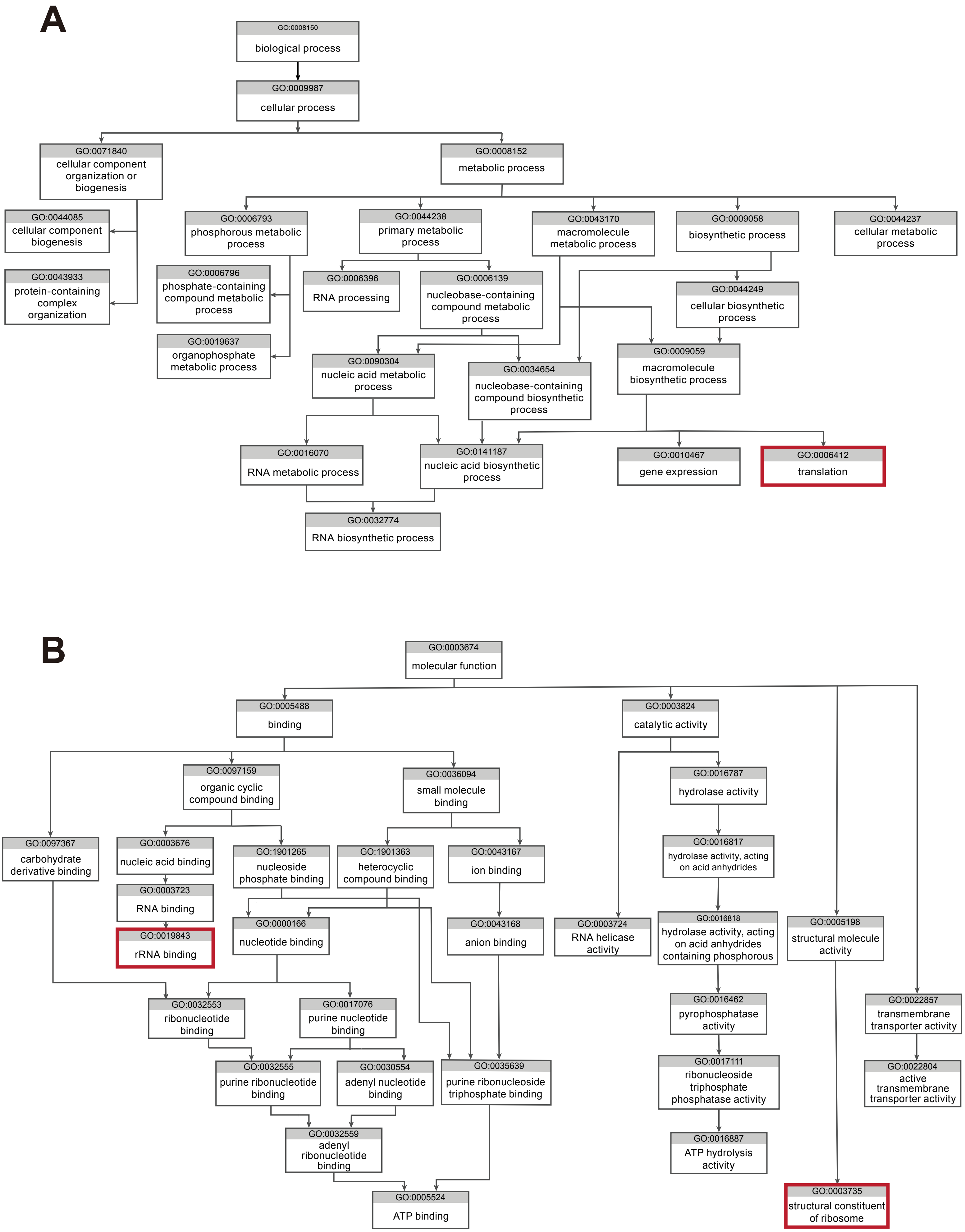
A
GO:0008150
biological process
GO:0009987
cellular process
GO:0071840
cellular component organization or biogenesis
GO:0008152
metabolic process
GO:0044085
cellular component biogenesis
GO:0043933
protein-containing complex organization
GO:0006793
phosphorous metabolic process
GO:0006796
phosphate-containing compound metabolic process
GO:0019637
organophosphate metabolic process
GO:0044238
primary metabolic process
GO:0006396
RNA processing
GO:0006139
nucleobase-containing compound metabolic process
GO:0043170
macromolecule metabolic process
GO:0009058
biosynthetic process
GO:0044237
cellular metabolic process
GO:0044249
cellular biosynthetic process
GO:0090304
nucleic acid metabolic process
GO:0034654
nucleobase-containing compound biosynthetic process
GO:0009059
macromolecule biosynthetic process
GO:0016070
RNA metabolic process
GO:0141187
nucleic acid biosynthetic process
GO:0010467
gene expression
GO:0006412
translation
GO:0032774
RNA biosynthetic process
B
GO:0003674
molecular function
GO:0005488
binding
GO:0003824
catalytic activity
GO:0097159
organic cyclic compound binding
GO:0036094
small molecule binding
GO:0016787
hydrolase activity
GO:0097367
carbohydrate derivative binding
GO:0003676
nucleic acid binding
GO:1901265
nucleoside phosphate binding
GO:1901363
heterocyclic compound binding
GO:0043167
ion binding
GO:0016817
hydrolase activity, acting on acid anhydrides
GO:0003723
RNA binding
GO:0019843
rRNA binding
GO:0000166
nucleotide binding
GO:0043168
anion binding
GO:0003724
RNA helicase activity
GO:0016818
hydrolase activity, acting on acid anhydrides containing phosphorous
GO:0005198
structural molecule activity
GO:0032553
ribonucleotide binding
GO:0017076
purine nucleotide binding
GO:0016462
pyrophosphatase activity
GO:0022857
transmembrane transporter activity
GO:0032555
purine ribonucleotide binding
GO:0030554
adenyl nucleotide binding
GO:0035639
purine ribonucleoside triphosphate binding
GO:0017111
ribonucleoside triphosphate phosphatase activity
GO:0022804
active transmembrane transporter activity
GO:0032559
adenyl ribonucleotide binding
GO:0016887
ATP hydrolysis activity
GO:0005524
ATP binding
GO:0003735
structural constituent of ribosome

Figure S3. Ribosome related gene ontology (GO) terms are consistently enriched in *E. coli*, *S. cerevisiae*, and *M. musculus*.

(A, B) Hierarchical tree graph displaying relationships between enriched GO terms in all three species, *E. coli*, *S. cerevisiae*, and *M. musculus* was sown. Each box represented the GO term and associated ID number. **(A)** Analysis of biological processes enriched GO terms shows 'translation' as one of the most specialized conserved GO categories. **(B)** Analysis of molecular function enriched GO terms shows 'rRNA binding' and 'structural constituent of ribosome' as two of the most specialized conserved categories.

Figure S4

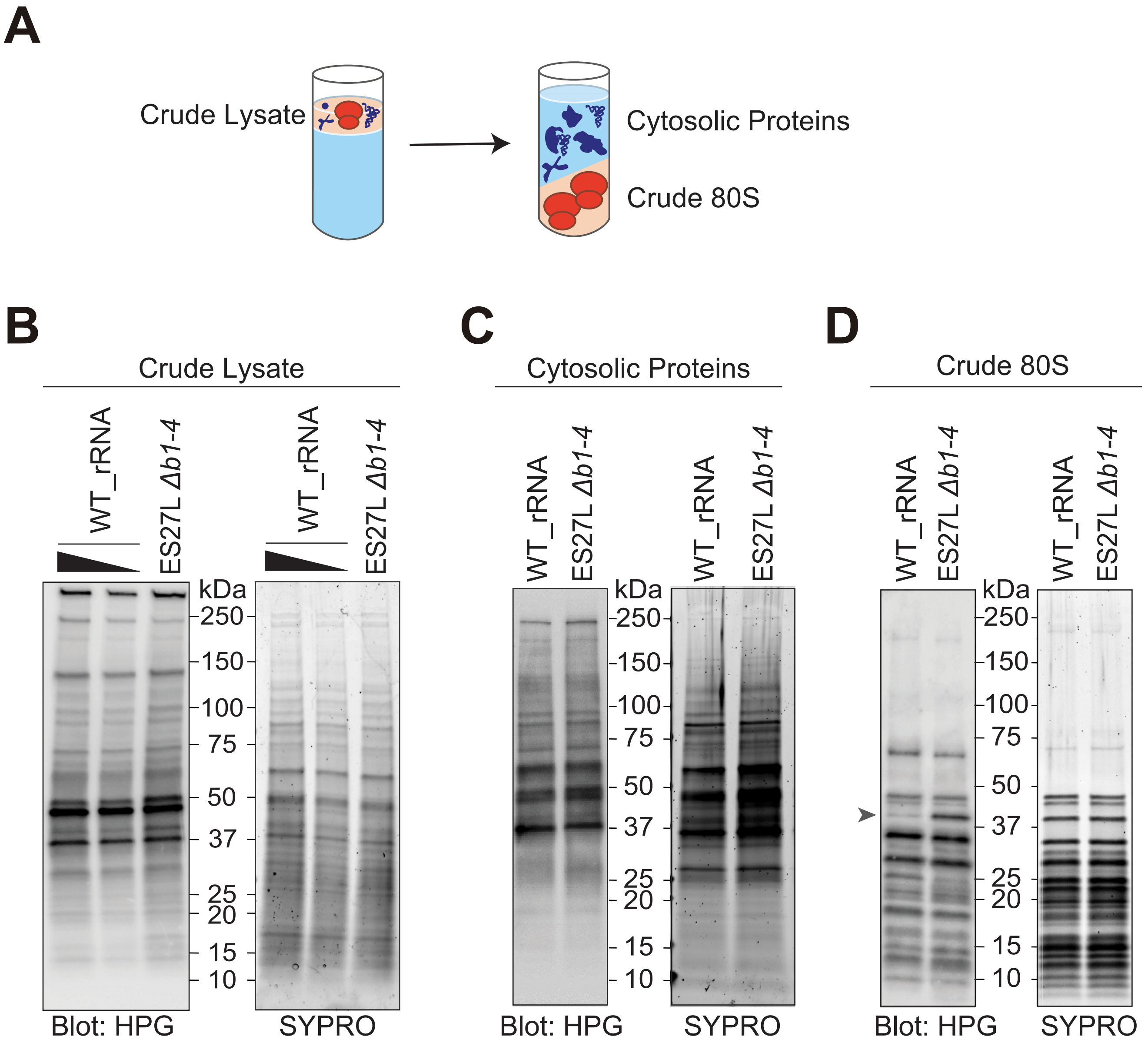
A
Crude Lysate
Cytosolic Proteins
Crude 80S
B
Crude Lysate
WT_rRNA
ES27L Δb1-4
kDa
250
150
100
75
50
37
25
20
15
10
Blot: HPG
SYPRO
C
Cytosolic Proteins
D
Crude 80S

Figure S4. HPG labeling does not show differences in crude lysate and cytoplasmic proteins between WT_rRNA and ES27L ∆*b1-4*, but shows a difference in crude 80S.

(A) The scheme of the experimental procedure indicates Crude Lysate (B) was fractionated by sucrose cushion to the cytoplasmic proteins in the supernatant (C) and a crude 80s ribosome pellet (D). **(B-D)** Strept-blot visualized HPG signals and SYPRO Ruby (SYPRO) staining visualized total protein. **(D)** A single band increased HPG signal in Crude 80S fraction from ES27L *Δb1-4* lysate.

Figure S5

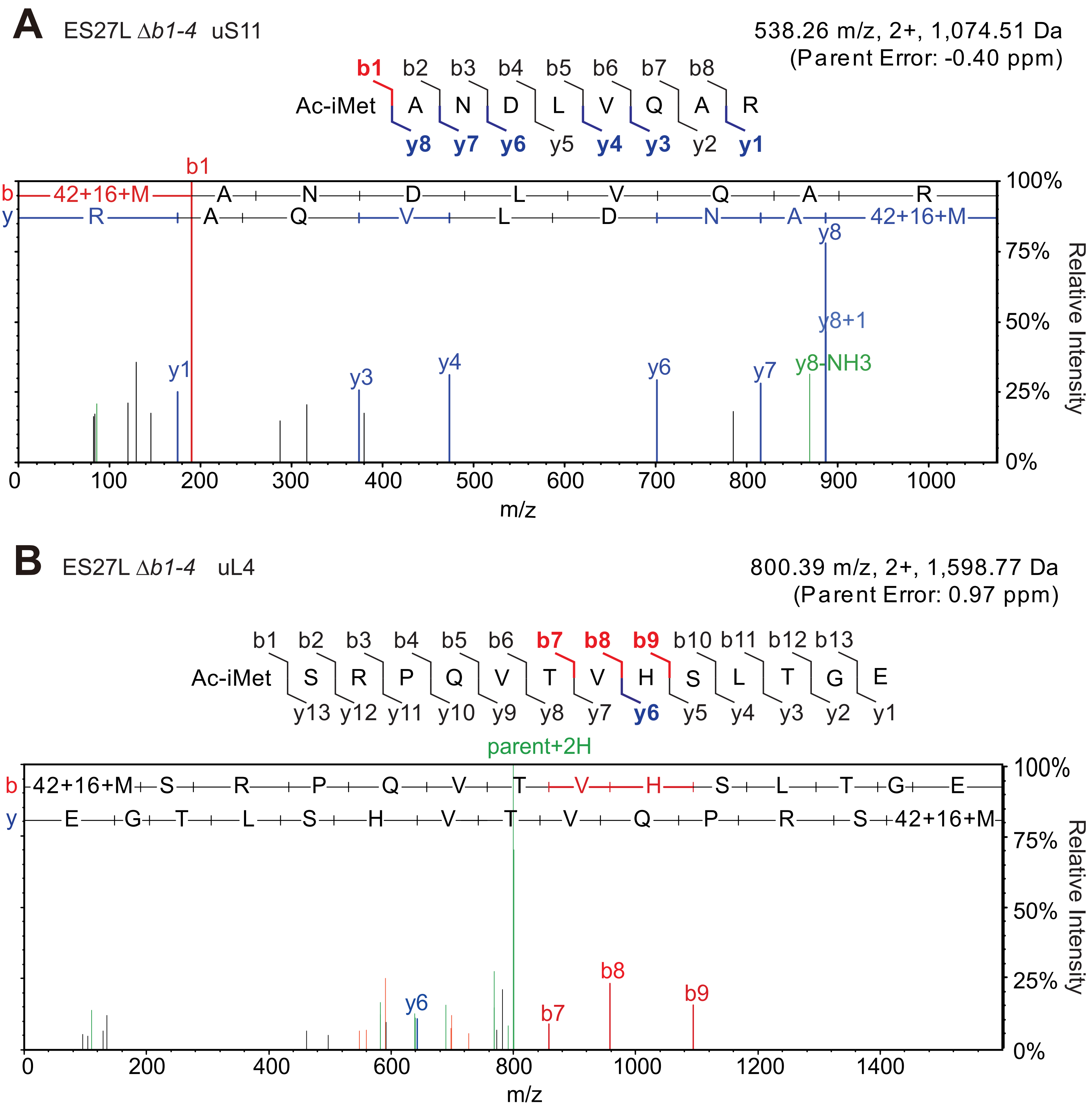
A
ES27L Δb1-4 uS11
538.26 m/z, 2+, 1,074.51 Da
(Parent Error: -0.40 ppm)
b1 b2 b3 b4 b5 b6 b7 b8
Ac-iMet A N D L V Q A R
y8 y7 y6 y5 y4 y3 y2 y1
b 42+16+M A N D L V Q A R
y R A Q V L D N A 42+16+M
b1
y1
y3
y4
y6
y7
y8-NH3
y8
y8+1
100%
75%
50%
25%
0%
Relative Intensity
0 100 200 300 400 500 600 700 800 900 1000
m/z
B
ES27L Δb1-4 uL4
800.39 m/z, 2+, 1,598.77 Da
(Parent Error: 0.97 ppm)
b1 b2 b3 b4 b5 b6 b7 b8 b9 b10 b11 b12 b13
Ac-iMet S R P Q V T V H S L T G E
y13 y12 y11 y10 y9 y8 y7 y6 y5 y4 y3 y2 y1
parent+2H
b 42+16+M S R P Q V T V H S L T G E
y E G T L S H V T V Q P R S 42+16+M
y6
b7
b8
b9
100%
75%
50%
25%
0%
Relative Intensity
0 200 400 600 800 1000 1200 1400
m/z

Figure S5. Detection of iMet retained peptides by Liquid Chromatography tandem Mass spectrometry (LC-MS/MS) from RPs in ES27L Δ*b1-4* ribosomes.

(A) Spectra of fragmented N-terminal peptide originating from uS11 protein purified from 40S subunit of ES27L Δ*b1-4* strain. Spectra detected Ac-iMet indicated iMet retention on uS11 in the ES27L Δ*b1-4* ribosomes. **(B)** N-terminal peptide from uL4 protein purified from 60S subunit of ES27L Δ*b1-4* strain. Spectra detected iMet containing b7, b8, and b9 fragments, which indicated iMet retention on uL4 in the ES27L Δ*b1-4* ribosomes.

Figure S6

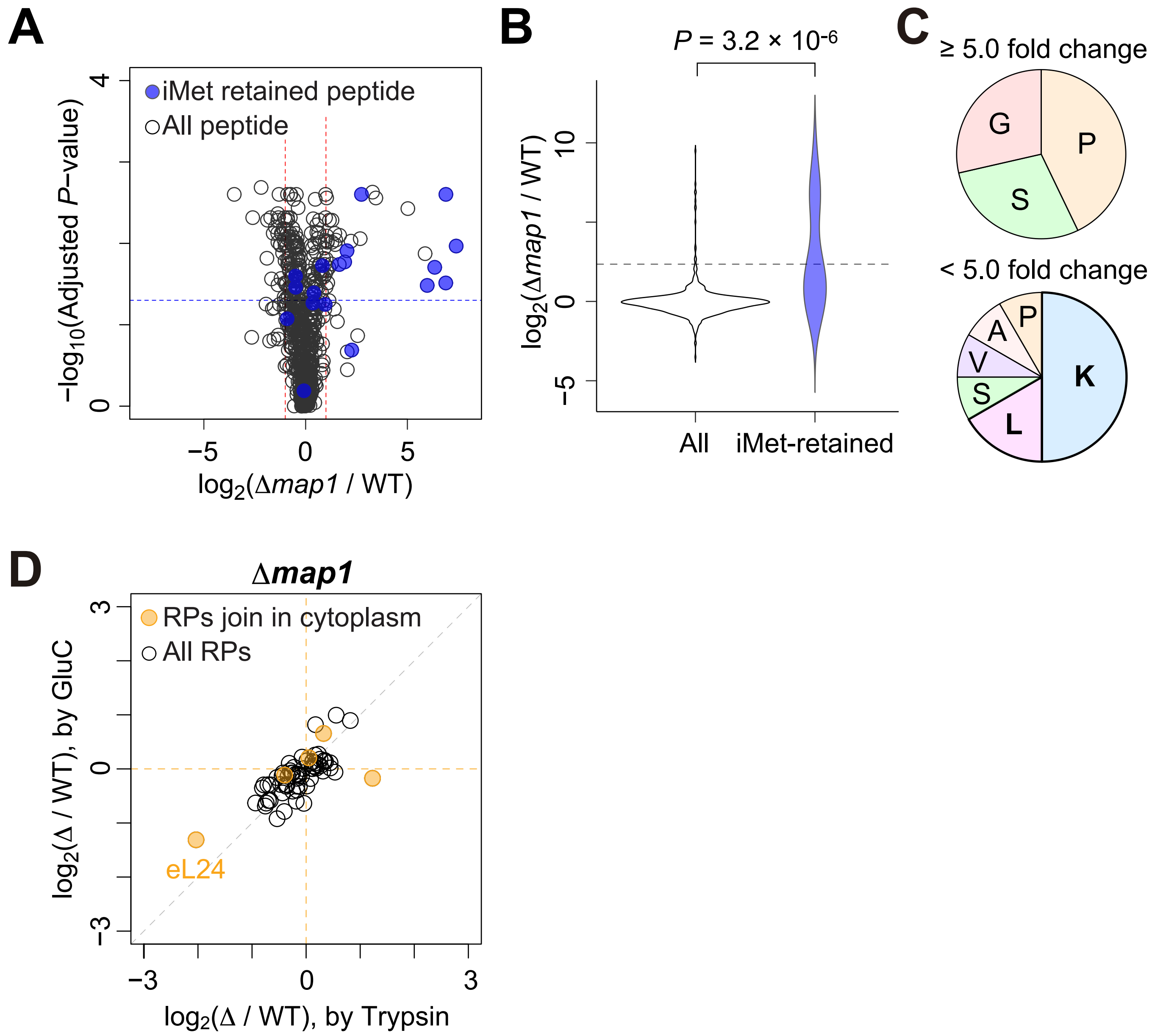
A
iMet retained peptide
All peptide
−log10(Adjusted P−value)
4
0
−5
0
5
log2(Δmap1 / WT)
B
P = 3.2 × 10-6
log2(Δmap1 / WT)
10
0
−5
All
iMet-retained
C
≥ 5.0 fold change
G
P
S
< 5.0 fold change
P
A
V
S
L
K
D
Δmap1
RPs join in cytoplasm
All RPs
3
0
−3
eL24
log2(Δ / WT), by GluC
−3
0
3
log2(Δ / WT), by Trypsin

Figure S6. Semi-quantitative proteomic analysis of crude ribosomes by nanoLC-MS/MS from ribosomes in Δ*map1* strain indicated changes in N-terminal peptide and in the composition of eL24 protein.

(A) A volcano plot of the peptides to show the fold changes between WT: W303 and Δ*map1* strains. Blue dots indicate iMet-retained peptides. The red dashed lines showed the lines x = ± $\log_2(5)$, and the blue dashed line showed the line y = $-\log_{10}(0.05)$. The *P*-values were determined by Welch's t-test (two-sided) and corrected by the Benjamini-Hochberg method. Deamidated and Oxidation peptides were omitted in this comparison. Fold change values and corresponding *P*-values adjusted by the Benjamini-Hochberg method were shown in Supplementary Dataset S12. **(B)** Distribution of the fold change of the peptides digested by GluC between WT: W303 and Δ*map1* strains represented as violin plots. A white graph indicated all quantified peptides, and a blue indicated iMet-retained peptides. The numbers of all peptides and iMet-retained peptides were 727 and 19, respectively. The *P*-value was obtained by the Wilcoxon rank sum test (two-sided). The dashed horizontal line indicates y = $\log_2(5)$ as the threshold used for Supplementary Figure S5F. **(C)** Distribution of second amino acids after first methionine for iMet-retained peptides. The iMet-retained peptides were divided into two groups by fold change values (more or less than five), and the distribution of the second amino acids for these two groups was shown. The total number of the iMet-retained peptides in each group is as follows: 7n for the ≥ 5-fold change group and 12 for the < 5-fold change group. **(D)** A scatter plot of fold changes of RPs between WT: W303 and Δ*map1* strains at a protein level. The median of the fold change values for each RP was used to depict the graph. The X-axis showed the fold changes when trypsin was used for peptide digestion, and the Y-axis showed those when GluC was used. Fold change values and corresponding *P*-values adjusted by the Benjamini-Hochberg method were listed in Supplementary Dataset S14.

Figure S7

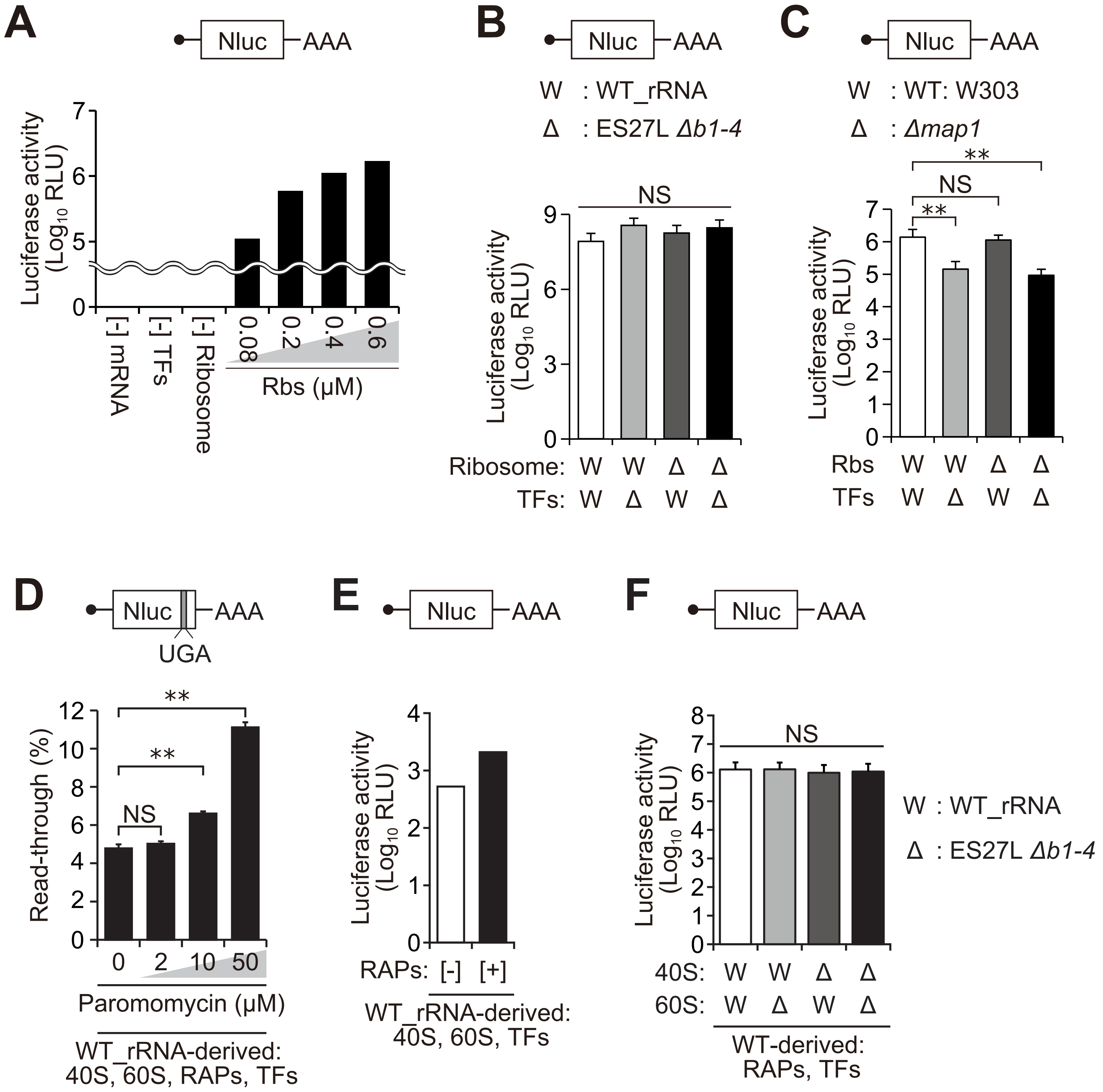
A
Nluc
AAA
Luciferase activity (Log10 RLU)
[-] mRNA
[-] TFs
[-] Ribosome
0.08
0.2
0.4
0.6
Rbs (μM)
B
W : WT_rRNA
Δ : ES27L Δb1-4
NS
Ribosome: W W Δ Δ
TFs: W Δ W Δ
C
W : WT: W303
Δ : Δmap1
**
NS
Rbs W W Δ Δ
TFs W Δ W Δ
D
UGA
Read-through (%)
Paromomycin (μM)
WT_rRNA-derived: 40S, 60S, RAPs, TFs
E
RAPs: [-] [+]
WT_rRNA-derived: 40S, 60S, TFs
F
40S: W W Δ Δ
60S: W Δ W Δ
WT-derived: RAPs, TFs

Figure S7. Establishing ribosome-switching IVT system.

(A) IVT translation activity in fractionated IVT lysate from WT: W303 was monitored using the WT Nluc mRNA without a stop codon. The reconstituted IVT system without mRNA, TFs, or ribosomes did not show any protein synthesis activity. Reconstituting the IVT system with more ribosomes increased IVT activity. **(B)** Translation activity of reconstituted IVT systems with all combinations of TFs and ribosomes between the WT_rRNA (W) and ES27L *Δb1-4* (Δ) strains. All combinations showed similar translation activity, indicating that ribosomes and TFs from the ES27L Δ*b1-4* strain have the same translation activity as the WT_rRNA strain. Data are presented as mean + SD (t-test, **p < 0.01; NS, not significant; n > 3). **(C)** Translation activity of ribosomes from the WT: W303 and *Δmap1* strains was monitored using the reconstituted IVT with TFs from the WT: W303 strain. TFs from the Δ*map1* strain showed much less translation activity. Data are presented as mean + SD (t-test, **p < 0.01; NS, not significant; n > 3). **(D)** Reconstituted IVT from purified subunits increased translation errors by paromomycin in a dose-dependent manner. Data are presented as mean + SD (t-test, **p < 0.01; NS, not significant; n > 3). **(E)** IVT reconstitution from purified 40S and 60S subunits provides active mRNA translation. Adding the RAPs fraction helped to increase translation activity. **(F)** Translation activity of reconstituted IVT from purified ribosome subunits. All combinations showed similar translation activity. Data are presented as mean + SD (t-test, **p < 0.01; NS, not significant; n > 3).

Supplementary Table S1. Yeast strains (*S. cerevisiae*) used in this paper

KAY488 (NOY890)	*MATA ura3-1 leu2-3,112 his3-11 trp1-1 ade2-1 can1-100 rdnaΔΔ::HIS3 carrying pRDN-hyg::URA3*	Laboratory of Katsura Asano (1)
WT_rRNA	KAY488: *MATA ura3-1 leu2-3,112 his3-11 trp1-1 ade2-1 can1-100 rdnaΔΔ::HIS3 carrying WT_rRNA::LEU2*	(2)
ES27L *Δb1-4*	KAY488: *MATA ura3-1 leu2-3,112 his3-11 trp1-1 ade2-1 can1-100 rdnaΔΔ::HIS3 carrying ES27L Δb1-4::LEU2*	(2)
W303-A	*MATA ura3-1 leu2-3,112 his3-11 trp1-1 ade2-1 can1-100*	Laboratory of Daniel Jarosz
Δmap1	W303-A: *MATA ura3-1 leu2-3,112 his3-11 trp1-1 ade2-1 can1-100 map1Δ0*	(2)
BY4741: WT	BY4741: *MATA ura3Δ0 leu2Δ0 his3Δ1 met15Δ0*	Laboratory of Daniel Jarosz
Δmap1	BY4741: *MATA ura3Δ0 leu2Δ0 his3Δ1 met15Δ0 map1Δ::*kan^r	Dharmacon
Δard1	BY4741: *MATA ura3Δ0 leu2Δ0 his3Δ1 met15Δ0 ard1Δ::*kan^r	Dharmacon
Δnat1	BY4741: *MATA ura3Δ0 leu2Δ0 his3Δ1 met15Δ0 nat1Δ::*kan^r	Dharmacon
Δnat5	BY4741: *MATA ura3Δ0 leu2Δ0 his3Δ1 met15Δ0 nat5Δ::*kan^r	Dharmacon
Δpdr5 (NBRP ID: BY25893)	*MATA Δpdr5::KanMX4 Δlys2::LEU2 ura3-1 leu2-3,112 his3-11,15 trp1-1 ade2-1*	National BioResource Project (NBRP), Japan (3)

1. Nemoto,N., Singh,C.R., Udagawa,T., Wang,S., Thorson,E., Winter,Z., Ohira,T., Ii,M., Valášek,L., Brown,S.J., *et al.* (2010) Yeast 18 S rRNA Is directly involved in the ribosomal response to stringent AUG selection during translation initiation. *Journal of Biological Chemistry*, 285, 32200–32212.
2. Fujii,K., Susanto,T.T., Saurabh,S. and Barna,M. (2018) Decoding the function of expansion segments in ribosomes. *Mol Cell*, 72, 1013–1020.
3. Saeki,Y., Kudo,T., Sone,T., Kikuchi,Y., Yokosawa,H., Toh-e,A. and Tanaka,K. (2009) Lysine 63-linked polyubiquitin chain may serve as a targeting signal for the 26S proteasome. *EMBO J*, 28, 359–371.

Supplementary Table S2. Plasmids used in this paper

pJD375: URA3, 2μ, Rluc-Fluc	Laboratory of Jonathan Dinman (4)
pJD375(MA): URA3, 2μ, Rluc-Fluc, Met-Ala	This paper
pJD375(ML): URA3, 2μ, Rluc-Fluc, Met-Lys	This paper
pJD433-UGA: URA3, 2μ, Rluc-UGA-Fluc	Laboratory of Jonathan Dinman (5)
pJD433-UGA(MA): URA3, 2μ, Rluc-UGA-Fluc, Met-Ala	This paper
pJD433-UGA(ML): URA3, 2μ, Rluc-UGA-Fluc, Met-Lys	This paper
pCMV-WT: CMV promoter, Rluc-Fluc	(2)
pCMV-433-UGA: CMV promoter, Rluc-UGA-Fluc	(2)
pNluc: pRS316: URA3, CEN, Flag-Nluc-HA	This paper
pNluc-UGA: pRS316: URA3, CEN, Flag-Nluc(UGA)-HA	This paper
pAla-Nluc: pRS316: URA3, CEN, Flag-Nluc-HA	This paper
pSer-Nluc: pRS316: URA3, CEN, Flag-Nluc-HA	This paper
pGly-Nluc: pRS316: URA3, CEN, Flag-Nluc-HA	This paper
pLys-Nluc: pRS316: URA3, CEN, Flag-Nluc-HA	This paper
pPgk1-Nluc: pRS316: URA3, CEN, Flag-Pgk1-Nluc-HA	This paper
pPgk1-Nluc(+1): pRS316: URA3, CEN, Flag-Pgk1-c-Nluc-HA	This paper
pPgk1-Nluc(-1): pRS316: URA3, CEN, Flag-Pgk1-cc-Nluc-HA	This paper

4. Muldoon-Jacobs,K.L.L. and Dinman,J.D. (2006) Specific effects of ribosome-tethered molecular chaperones on programmed-1 ribosomal frameshifting. *Eukaryot Cell*, 5, 762.

5. Harger,J.W. and Dinman,J.D. (2004) Evidence against a direct role for the Upf proteins in frameshifting or nonsense codon readthrough. *RNA*, 10, 1721–1729.

Supplementary Table S3. Sequence of DNA templates for in vitro translation

WT_Nluc	AGTACTTAATACGACTCACTATAGGGAATTCACATTTGCTTCTGACACAACTGTGTTCACTAGCAACCTCAAACAGACACCatggattacaaagacgacgacgataaaAGTAGCGGCGGAGGT**ATGGTCTTCACACTCGAAGATTTCGTTGGGGACTGGCGACAGACAGCCGGCTACAACCTGGACCAAGTCCTTGAACAGGGAGGTGTGTCCAGTTTGTTTCAGAATCTCGGGGTGTCCGTAACTCCGATCCAAAGGATTGTCCTGAGCGGTGAAAATGGGCTGAAGATCGACATCCATGTCATCATCCCGTATGAAGGTCTGAGCGGCGACCAAATGGGCCAGATCGAAAAAATTTTTAAGGTGGTGTACCCTGTGGATGATCATCACTTTAAGGTGATCCTGCACTATGGCACACTGGTAATCGACGGGGTTACGCCGAACATGATCGACTATTTCGGACGGCCGTATGAAGGCATCGCCGTGTTCGACGGCAAAAAGATCACTGTAACAGGGACCCTGTGGAACGGCAACAAAATTATCGACGAGCGCCTGATCAACCCCGACGGCTCCCTGCTGTTCCGAGTAACCATCAACGGAGTGACCGGCTGGCGGCTGTGCGAACGCATTCTGGCG**tatccctacgatgttcccgattacgcaTAA<br>(Single underline: T7 promoter / Double underline: 5’ UTR of human beta-globin / Small letters: FLAG, HA / Bold: Nluc)
WT_Pgk1-Nluc	ATCCGTAATACGACTCACTATAGGGTAACCGAGCTCGGACTatggattacaaagacgacgacgacaaaGAATTATCTTCAAAGTTGTCTGTCCAAGATTTGGACTTGCAGGACAAGCGTGTCTTCATCAGAGTTGCCTTCAACGTCCCATTGGACGGTACGAAGATC**ACGGTCTTCACACTCGAAGATTTCGTTGGGGACTGGCGACAGACAGCCGGCTACAACCTGGACCAAGTCCTTGAACAGGGAGGTGTGTCCAGTTTGTTTCAGAATCTCGGGGTGTCCGTAACTCCGATCCAAAGGATTGTCCTGAGCGGTGAAAATGGGCTGAAGATCGACATCCATGTCATCATCCCGTATGAAGGTCTGAGCGGCGACCAAATGGGCCAGATCGAAAAAATTTTTAAGGTGGTGTACCCTGTGGATGATCATCACTTTAAGGTGATCCTGCACTATGGCACACTGGTAATCGACGGGGTTACGCCGAACATGATCGACTATTTCGGACGGCCGTATGAAGGCATCGCCGTGTTCGACGGCAAAAAGATCACTGTAACAGGGACCCTGTGGAACGGCAACAAAATTATCGACGAGCGCCTGATCAACCCCGACGGCTCCCTGCTGTTCCGAGTAACCATCAACGGAGTGACCGGCTGGCGGCTGTGCGAACGCATTCTGGCG**tatccctacgatgttcccgattacgcaTAA<br>(Single underline: T7 promoter / Double underline: 5’ UTR derived from multiple cloning site of pYES2 plasmid / Small letters: FLAG, HA / Gray-shaded: Pgk1 /Bold: Nluc)

Supplementary Table S4. Sequence of primers for PCR

Primer1	AGTACTTAATACGACTCACTATAGGGAATTCACATTTGCTTCTGACACAACTGTGTTCACTAGCAACCTCAAACAGACACCatggattacaaagacgacgacgata<br>(Single underline: T7 promoter / Double underline: 5' UTR of human beta-globin / Small letters: FLAG)
Primer2	TTAtgcgtaatcgggaacatcgtaggga<br>(Small letters: HA)
Primer3	ATCCGTAATACGACTCACTATAGGGTAACCGAGCTCGGACTatggattacaaagacgacgacgaca<br>(Single underline: T7 promoter / Double underline: 5' UTR derived from multiple cloning site of pYES2 plasmid / Small letters: FLAG)
25S fwd	CCAAGGAGTCTAACGTCTATGCGAGTG
25S rev	AGAGTTTCCTCTGGCTTCAC
Pre-25S fwd	CTAGGCGAACAATGTTCTTAAAG
Pre-25S rev	GATTCTCACCCTCTATGACG
18S fwd	ACATCCAAGGAAGGCAGCAG
18S rev	AATATACGCTATTGGAGCTGGAATTACCG
Pre-18S fwd	ACTCCATCTCAGAGCGGAGA
Pre-18S rev	CCATCTCTTGTCTTCTTGCC

Supplementary Table S5. Enriched GO terms for *Escherichia Coli* (REFLIST 4403)

Supplementary Table S6. Enriched GO terms for *Saccharomyces Cerevisiae* (REFLIST 6060)

Supplementary Table S7. Enriched GO terms for *Mus Musculus* (REFLIST 21836)

Supplementary Table S8. Ribosomal Proteins reference list

Supplementary Table S9. TMT-MS data from (Fujii et al., 2018)

Supplementary Table S10. List of iMet-retaining peptides increased in mutant ribosomes

Supplementary Table S11. ES27L Δ*b1-4* peptide data set

Supplementary Table S12. Δ*map1* peptide data set

Supplementary Table S13. ES27L Δ*b1-4* protein data set

Supplementary Table S14. Δ*map1* protein data set
